# Alternative ribosomal protein RpmE2 is produced under zinc limitation in *Neisseria gonorrhoeae* and slows translation and bacterial growth

**DOI:** 10.1128/mbio.01137-26

**Published:** 2026-06-18

**Authors:** Amy L. Forehand, Kinga Malezyna, Keena S. Thomas, Ian J. Glomski, Cynthia Nau Cornelissen, Ahmad Jomaa, Alison K. Criss

**Affiliations:** 1 Department of Microbiology, Immunology, and Cancer Biology, University of Virginia 174484, Charlottesville, Virginia, USA; 2 Department of Molecular Physiology and Biological Physics, University of Virginia 174481, Charlottesville, Virginia, USA; 3 Institute for Biomedical Sciences, Georgia State University 439338, Atlanta, Georgia, USA; NYU Langone Health, New York, New York, USA

**Keywords:** *Neisseria gonorrhoeae*, zinc, ribosome, translation, nutritional immunity

## Abstract

To enhance its pathogenic potential, *Neisseria gonorrhoeae* (Gc) pirates zinc from human metal sequestration proteins using TonB-dependent outer membrane transport systems. However, cytoplasmic mechanisms by which Gc adapts to zinc limitation are still uncharacterized. *rpmE2* and *rpmJ2* transcripts, encoding alternative L31 and L36 ribosomal proteins, respectively, which are not predicted to bind zinc, are significantly more abundant in zinc-limited Gc. In other bacterial species, alternative ribosomal proteins replace canonical zinc-binding ribosomal proteins when zinc availability is low, enabling growth under metal limitation. We found that Gc *rpmE2* and *rpmJ2* are in an operon and transcriptionally induced under zinc limitation by derepression via the zinc uptake regulator Zur, while genes encoding canonical proteins *rpmE* and *rpmJ* are not zinc-regulated. In contrast to other bacteria, ribosomes in zinc-limited Gc contained both RpmE2 and RpmE. Furthermore, Gc deleted for RpmE2 and RpmJ2 grew better than the wild-type parent under zinc limitation. Gc engineered to produce only RpmE2 exhibited a growth defect relative to RpmE-only Gc, regardless of zinc availability. Moreover, ribosomes from RpmE2-only Gc had reduced translation *in vitro*. Thus, unlike other bacteria, the alternative L31 protein RpmE2 is not functionally equivalent to the canonical RpmE in Gc. Instead, the ribosomes of zinc-limited Gc are heterogeneous, containing either alternative or canonical ribosomal proteins. We propose that L31 ribosomal protein alternation allows Gc to withstand zinc limitation by reducing translation and slowing growth, to prolong its survival when encountering host-imposed nutritional immunity.

**IMPORTANCE:**

Zinc acquisition is crucial for growth and infectivity of *Neisseria gonorrhoeae* (Gc). However, research on Gc adaptation to zinc limitation has primarily focused on outer and inner membrane zinc transporters. Here, we implicate ribosomal protein alternation in the Gc response to zinc limitation. Ribosomes containing the non-zinc-binding, alternative ribosomal protein RpmE2 are less translationally active, and RpmE2-producing bacteria grow more slowly. This contrasts with reports from other bacteria, where ribosomal protein alternation facilitates growth in zinc-limited conditions. This work uncovers a new way in which ribosomal protein alternation enables bacterial resistance to nutritional immunity.

## INTRODUCTION

*Neisseria gonorrhoeae* (Gc) is an obligate human pathogen and the causative agent of the sexually transmitted infection gonorrhea. The CDC has designated gonorrhea as an urgent public health threat due to increasing antibiotic resistance and serious clinical sequelae associated with Gc infection (1). Untreated gonorrhea can cause pelvic inflammatory disease, epididymitis, and infertility (2–4). Phase and antigenic variation of immunogenic Gc surface structures prevents natural protective immunity from developing and has hindered vaccine development, while Gc infection skews towards a type 17, neutrophilic immune response that is associated with eliciting inflammatory damage (5–7).

Acquisition of essential metals, including zinc, is crucial for Gc pathogenesis. Zinc is present in ~5% of the bacterial proteome (8), but zinc availability at Gc infection sites varies. Zinc sequestration proteins calprotectin (~1 µM [9]) and psoriasin (~62 nM [10]) are abundant in the female reproductive tract (11). In contrast, zinc is abundant in seminal fluid (~1–3 mM) (12, 13) and in phagosomes (14). Understanding how Gc maintains zinc homeostasis during infection will broaden understanding of Gc pathogenesis and inform therapeutic development.

Bacterial ribosomes can be a major intracellular zinc source. In *Escherichia coli*, each ribosome binds ~8 equivalents of zinc (15, 16). Bacterial ribosomes contain 50S and 30S subunits, comprised of 23S, 5S, and 16S rRNAs and over 50 ribosomal proteins. In some bacterial genomes, one or more genes encoding zinc-binding, canonical ribosomal proteins have undergone duplication and evolution to yield non-zinc-binding, alternative paralogous proteins (17). Predicted zinc-binding paralogs encode a cysteine zinc-binding motif absent from alternative paralogs (17). Duplicated ribosomal proteins vary among bacterial species and include L28, L31, L33, L36, S14, and S18. In these species, alternative ribosomal proteins are produced under low zinc conditions and regulated by Zur (18–25). Zinc-bound Zur binds palindromic regions of DNA known as Zur boxes in the genes it regulates, repressing their transcription when zinc is replete. Zur loses affinity for the Zur box when zinc is limited, and Zur-regulated genes are derepressed (26).

Several hypotheses exist about Zur-regulated alternative ribosomal proteins in zinc-limited bacteria. In *Bacillus subtilis*, alternative L31 is produced under zinc limitation, and canonical L31 is displaced from the ribosome and degraded; this is hypothesized to liberate zinc for other cellular uses (27). In several bacterial species, the loss of alternative ribosomal proteins leads to a growth defect when zinc is limited (23, 24, 28–31). Ribosomal protein paralogs also alter translation (32). Ribosomes containing alternative proteins can exhibit reduced translation efficiency, including reduced processivity and increased frameshifting (33). Zinc-limited mycobacterial ribosomes recruit hibernation factors that stabilize and inactivate the ribosome (30, 34), although the role of non-zinc-binding ribosomal proteins in this process is debated (35). In other reports, alternative ribosomes are equally translationally active as ribosomes containing canonical paralogs (19, 21, 32, 35).

**Fig 1:**
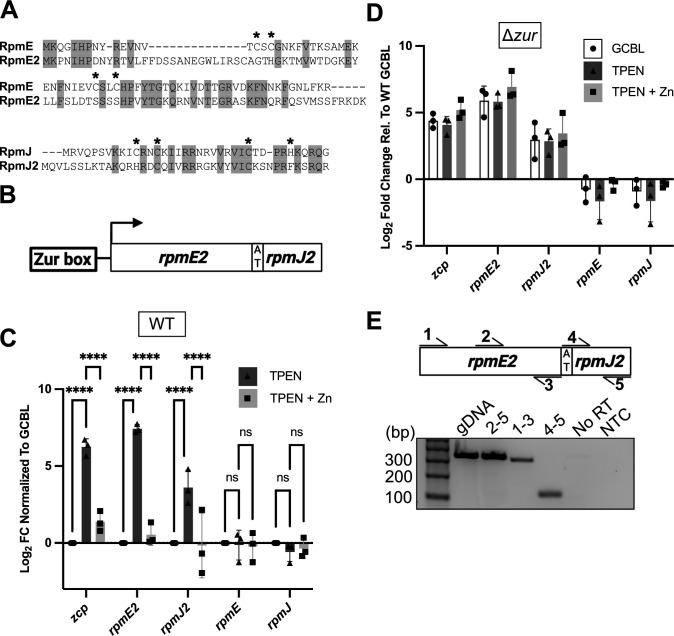
The *rpmE2-rpmJ2* operon is zinc-repressed via Zur, while *rpmE* and *rpmJ* are not zinc-regulated. (**A**) Amino acid sequences of FA1090 RpmE and RpmE2 and RpmJ and RpmJ2 were aligned in UniProt (42). Residues highlighted in gray are conserved between the paralogs, and asterisks indicate the residues predicted to participate in zinc-binding in RpmE and RpmJ (17). (**B**) Schematic of the *rpmE2rpmJ2* operon, including the Zur binding site. “AT” represents the overlapping base pair of the *rpmE2* and *rpmJ2* open reading frames. (**C**) WT Gc was inoculated into modified GCBL, modified GCBL + 20 µM TPEN, or modified GCBL + 20 µM TPEN + 10 µM ZnSO_4_ and cultured to mid-log phase (*T* = 3 h). RNA was extracted and analyzed by qRT-PCR (2^−ΔΔCt^) with normalization to *ngo1823* (encodes L15 ribosomal protein) in untreated media. (**D**) FA1090 WT and Δ*zur* Gc were cultured and RNA extracted as in panel **B**. RNA was analyzed by qRT-PCR (2^−ΔΔCt^) with normalization to 5S rRNA in WT Gc in untreated media. *zcp* is a positive control for a zinc- and Zur-regulated transcript (43). (**E**) WT Gc was inoculated into modified GCBL + 20 µM TPEN as in panel **C**. RNA was extracted and subjected to RT-PCR with primers overlapping *rpmE2-rpmJ2* (2, 5) or spanning *rpmE2* (1, 3) or *rpmJ2* (4, 5) individually. Primer binding sites relative to *rpmE2* and *rpmJ2* are indicated in the schematic. PCR amplicon was analyzed on a 2% agarose gel. Genomic DNA (gDNA, amplified with primers 2 and 5) served as positive control, and negative controls were no reverse transcriptase (RT) and no DNA template (NTC). The gel in panel **E** is representative of one of three biological replicates. Each symbol in panels C and D is representative of one biological replicate ± standard deviation from the mean; asterisks represent the adjusted *P*-values by two-way ANOVA and Tukey’s multiple comparisons test. Comparisons were made within each primer set, ****P < 0.0001, ns = not significant; comparisons not shown are not significant.

Pathogenic *Neisseria* encode two paralogous L31 ribosomal proteins and two paralogous L36 ribosomal proteins (17) (Fig. 1A). *rpmE* (NGO_2126, NEIS1928 [36] in strain FA1090) encodes the canonical L31 ribosomal protein. In other bacteria, RpmE bridges the 50S and 30S subunits and maintains ribosome stability during translation (37, 38). *rpmJ* (NGO_09710, NEIS0154) encodes the canonical L36 ribosomal protein and binds 23S rRNA (39). Gc alternative L31 and L36 ribosomal proteins RpmE2 (NGO_0930, NEIS0920) and RpmJ2 (NGO_0931, NEIS0919) are predicted to be in a Zur-regulated operon (40, 41) (Fig. 1B). *rpmE* and *rpmJ* are not predicted to be in an operon or regulated by Zur, and these ORFs are not proximal to each other or to *rpmE2* and *rpmJ2* in the Gc genome.

We and others reported that *rpmE2* is upregulated in pathogenic *Neisseria* grown under conditions where zinc is not bioavailable (40, 44) and in a *zur* mutant (41). Here, we examined how *rpmE2* and *rpmJ2* are regulated in and affect growth of zinc-limited Gc. The *rpmE2-rpmJ2* operon was induced in Gc under zinc limitation via Zur derepression. *rpmE* and *rpmJ* expression was independent of zinc availability and Zur. RpmE levels did not significantly change in zinc-limited Gc, in contrast to other bacteria (27). Rather, both RpmE and RpmE2 were present in ribosomes from zinc-limited Gc. Surprisingly, Gc lacking RpmE2 and RpmJ2 survived zinc limitation better than WT bacteria, containing both canonical and alternative ribosomal proteins. Gc engineered to produce RpmE2 as its only L31 protein exhibited a growth defect relative to Gc only producing RpmE, regardless of zinc availability. Furthermore, ribosomes purified from RpmE2-only Gc had reduced translational capacity *in vitro* relative to RpmE-only ribosomes. We conclude that as part of the Gc response to zinc limitation, the alternative ribosomal protein RpmE2 incorporates into ribosomes, where it slows translation and consequent bacterial growth.

## RESULTS

### The *rpmE2-rpmJ2* operon is zinc-repressed via Zur; *rpmE* and *rpmJ* are not zinc-regulated

To assess expression of Gc *rpmE2*, *rpmE*, *rpmJ2*, and *rpmJ*, RNA was collected from zinc-limited and zinc-replete Gc, and transcript abundance was analyzed by qRT-PCR. Gc were grown in gonococcal base liquid medium (GCBL), GCBL containing N,N,N′,N′-tetrakis(2-pyridinylmethyl)-1,2-ethanediamine (TPEN), a chelator with high affinity for zinc, or GCBL containing TPEN and zinc (ZnSO_4_). The concentrations of TPEN and zinc used in all experiments were empirically determined to fully repress or induce production of Zur-regulated gene products, respectively, and adjusted with different GCBL batches. *rpmE2* and *rpmJ2* transcripts were significantly increased in zinc-limited Gc compared with Gc in untreated GCBL, and abundance significantly decreased with addition of zinc (Fig. 1C). In contrast, *rpmE* and *rpmJ* expression did not significantly change between conditions (Fig. 1C).

To evaluate Zur-mediated regulation of Gc L31 and L36 transcripts, expression of *rpmE, rpmE2, rpmJ*, and *rpmJ2* was measured by qRT-PCR in *zur* mutant (Δ*zur*) Gc as above. *rpmE2* and *rpmJ2* transcripts were elevated in Δ*zur* Gc relative to untreated WT bacteria and were not repressed with excess zinc (Fig. 1D). Transcript abundance of *rpmE* and *rpmJ* was unaffected by loss of *zur* (Fig. 1D). Thus, *rpmE2* and *rpmJ2* are Zur-repressed in Gc. *rpmE2* and *rpmJ2* ORFs overlap by one base pair (Fig. 1B). To test if *rpmE2* and *rpmJ2* are co-transcribed, RNA from zinc-limited Gc was examined by RT-PCR for amplification of a region spanning the ORFs (Fig. 1E). An amplicon corresponding to the *rpmE2-rpmJ2* overlap was detected, indicating they are co-transcribed (Fig. 1E). Altogether, we conclude that *rpmE2-rpmJ2* constitute an operon that is induced in zinc-limited Gc by Zur derepression, while *rpmE* and *rpmJ* are not zinc- or Zur-regulated.

### Both RpmE2 and RpmE are produced and incorporated into ribosomes in zinc-limited Gc

**Fig 2:**
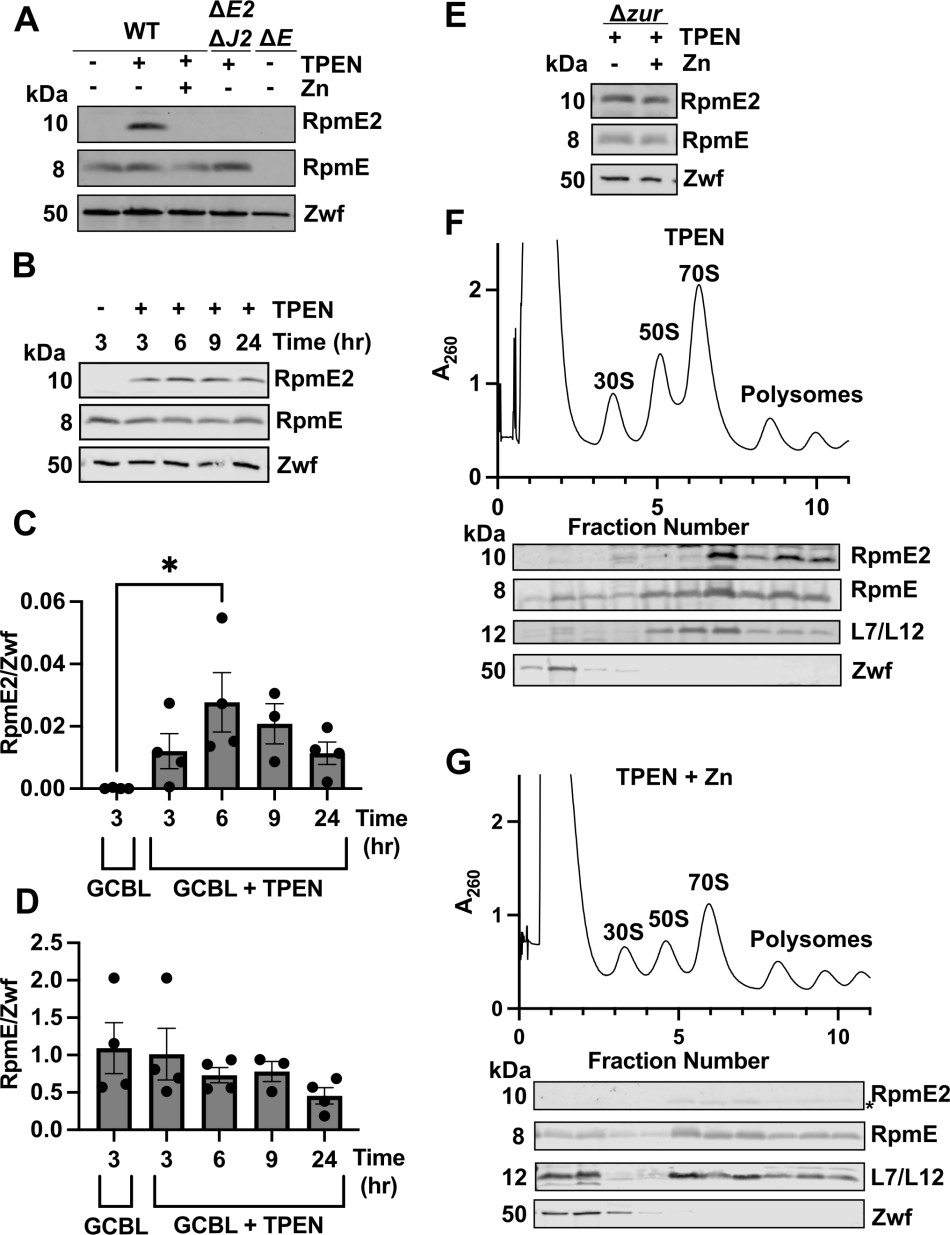
Both RpmE2 and RpmE are produced and incorporated into ribosomes in zinc-limited Gc. WT, Δ*rpmE2*Δ*rpmJ2*, or Δ*rpmE* (**A**) Gc was inoculated into modified GCBL, modified GCBL + 20 µM TPEN, and modified GCBL + 20 µM TPEN + 10 µM ZnSO_4_ as indicated. At 3, 6, 9, and 24 h, bacterial aliquots were taken and prepared for whole cell lysates, and Gc was diluted into fresh media. Lysates in panel **A** were collected at *T* = 6 h. Lysates were separated by 4%–16.5% Tris-Tricine SDS-PAGE, transferred to nitrocellulose, and immunoblotted for RpmE2, RpmE, and Zwf (loading control). (**B**) Whole cell lysates from 3, 6, 9, and 24 h post-inoculation into treated media were immunoblotted for RpmE2, RpmE, and Zwf. (**C and D**) Quantification of band intensity for RpmE2 and RpmE at the indicated time points in panel B was normalized to Zwf band intensity. Each symbol is representative of one biological replicate, *N* = 3–4, bars represent the mean ± SEM. Statistics represent the results of an ordinary one-way ANOVA with Šídák’s multiple comparisons test for GCBL at 3 h relative to other conditions. *P < 0.05, comparisons not shown are not significant. (**E**) Δ*zur* Gc was cultured for 6 h in modified GCBL with 20 μM TPEN and with or without 10 μM ZnSO._4._ Whole cell lysates were collected and immunoblotted for RpmE2, RpmE, and Zwf, as described for panels **A and B**. (**F and G**) Clarified cell extracts from WT Gc cultured to mid-log phase in modified GCBL + 25 µM TPEN (**F**) or modified GCBL + 20 µM TPEN + 10 µM ZnSO_4_ (**G**) were applied to a 10%–40% sucrose gradient and subjected to ultracentrifugation, followed by fractionation with UV monitoring. Each trace represents one biological replicate. Equal volumes of TCA-precipitated fractions were separated by SDS-PAGE and immunoblotted for RpmE2, RpmE, L7/L12, and Zwf. The asterisk in panel **G** represents RpmE background in the RpmE2 channel. The immunoblots in panels A, B, and E–G are representative of one of at least three biological replicates (see Fig. S1 for immunoblots of additional polysome profile replicates).

To examine RpmE and RpmE2 protein levels in Gc, whole cell lysates were prepared from Gc grown in untreated GCBL, GCBL with TPEN, or GCBL with TPEN and zinc. TPEN was added to a concentration (20 μM) that permitted Gc growth while strongly inducing RpmE2. RpmE2 and RpmE were detected with affinity-purified anti-peptide antibodies; Zwf (gonococcal glucose-6-phosphate-1-dehydrogenase) served as a loading control (45). Lysates from Δ*rpmE2*Δ*rpmJ2* and Δ*rpmE* Gc served as negative controls for RpmE2 and RpmE antibody reactivity, respectively. At 6 h, RpmE2 was produced only by zinc-limited Gc, while RpmE was produced by Gc in all conditions (Fig. 2A). RpmJ2 and RpmJ were undetectable by immunoblot.

Lysates collected at 3, 6, 9, and 24 h post-TPEN addition were used to assess RpmE2 and RpmE production by WT Gc over time. RpmE2 was produced as early as 3 hours post-TPEN addition, reached a maximum at 6 h, and remained steady thereafter (Fig. 2B and C). Notably, total RpmE levels did not significantly change over time and were no different from Gc grown in zinc-available conditions (Fig. 2B and D). In Δ*zur* Gc, both RpmE and RpmE2 were produced regardless of zinc availability (Fig. 2E).

To examine how zinc availability affects incorporation of RpmE and RpmE2 into ribosomes, Gc was grown to mid-logarithmic phase in zinc-limited (GCBL with TPEN) or zinc-replete (GCBL with TPEN and zinc) conditions, at concentrations that were empirically determined to induce or repress RpmE2 in each biological replicate. Clarified Gc lysates were separated over a sucrose density gradient by ultracentrifugation and fractionated to separate 30S and 50S ribosomal subunits, 70S ribosomes, and polysomes (mRNA-bound ribosomes). In zinc-limited Gc, RpmE2 was detected in 70S ribosome and polysome fractions (Fig. 2F; Fig. S1A and B). RpmE was detected in 70S ribosomes and polysomes from zinc-limited Gc, and also in 50S subunit fractions (Fig. 2F; Fig. S1A and B). In zinc-replete Gc, RpmE was detected in 50S subunit fractions, 70S ribosomes, and polysomes, while RpmE2 was not detected in any fraction (Fig. 2G; Fig. S1C and D).

Together, these data show that RpmE2 is made by zinc-limited Gc treated with TPEN and incorporated into ribosomes, but RpmE is not fully replaced by RpmE2 under these conditions. We propose that the paradigm for loss of canonical ribosomal proteins under zinc limitation, as reported in other bacteria, does not apply to Gc.

### Gc lacking RpmE2 and RpmJ2 are less susceptible to zinc limitation

**Fig 3:**
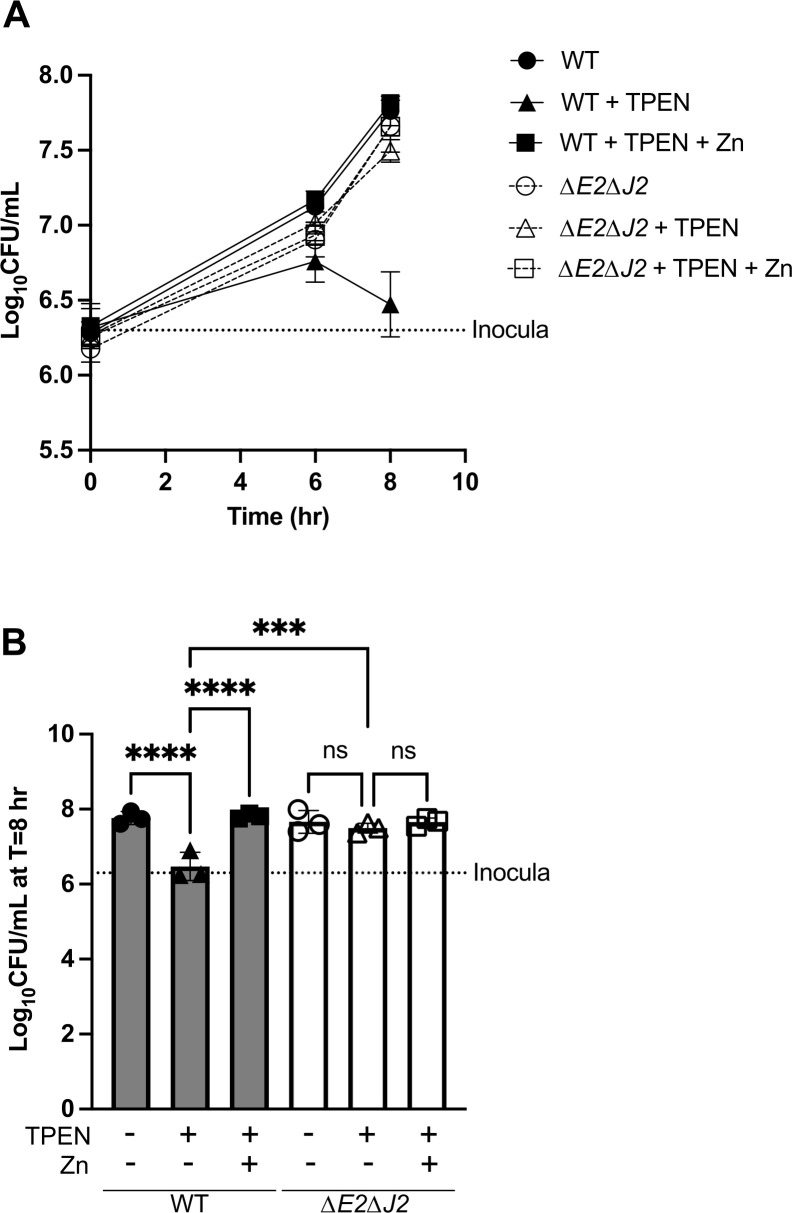
Gc lacking RpmE2 and RpmJ2 are less susceptible to growth restriction when zinc is limited. (**A**) WT or Δ*E2*Δ*J2* Gc (2 × 10^6^ CFU) was inoculated into Chelex-treated chemically defined medium (CDM) with 1 μM Fe(NO_3_)_3_, and with or without 0.25 μM TPEN and 2.5 μM ZnSO_4_. At 0, 6, and 8 h post-inoculation, 20 μL of culture was removed and serially diluted to enumerate CFU/mL. Data represent the mean of three biological replicates ± the standard error of the mean. (**B**) Log_10_CFU/mL for WT and Δ*E2*Δ*J2* Gc at *T* = 8 h. Each symbol represents a biological replicate ± standard deviation from the mean (three biological replicates). Asterisks represent the adjusted *P*-values calculated by ordinary one-way ANOVA followed by Šídák’s multiple comparisons test, ****P < 0.0001, ***P < 0.001, ns = not significant. Comparisons not shown are not significant.

To examine *rpmE2* and *rpmJ2* in growth of zinc-limited Gc, the *rpmE2-rpmJ2* operon was deleted and replaced with a kanamycin resistance cassette (Δ*E2*Δ*J2*). WT and Δ*E2*Δ*J2* Gc were grown in Chelex-treated chemically defined medium (CDM), CDM with 0.25 μM TPEN, or CDM with 0.25 μM TPEN and 2.5 μM zinc. CDM was used to limit contaminant metal and standardize metal-dependent growth. WT and Δ*E2*Δ*J2* Gc grew similarly in untreated CDM and CDM with TPEN and zinc, increasing ~1 log over 8 h (Fig. 3A and B). Surprisingly, in TPEN-containing CDM, significantly fewer WT Gc were recovered at 8 h than Δ*E2*Δ*J2* bacteria (Fig. 3B). While TPEN addition to CDM reduced growth of WT Gc at 8 h, compared with the other conditions, Δ*E2*Δ*J2* Gc grew equivalently regardless of zinc availability (Fig. 3B). We conclude that unlike other bacterial species, Gc producing alternative ribosomal proteins RpmE2 and RpmJ2 grow less well when zinc is limited.

### RpmE2 and RpmE exhibit structural and functional differences

**Fig 4:**
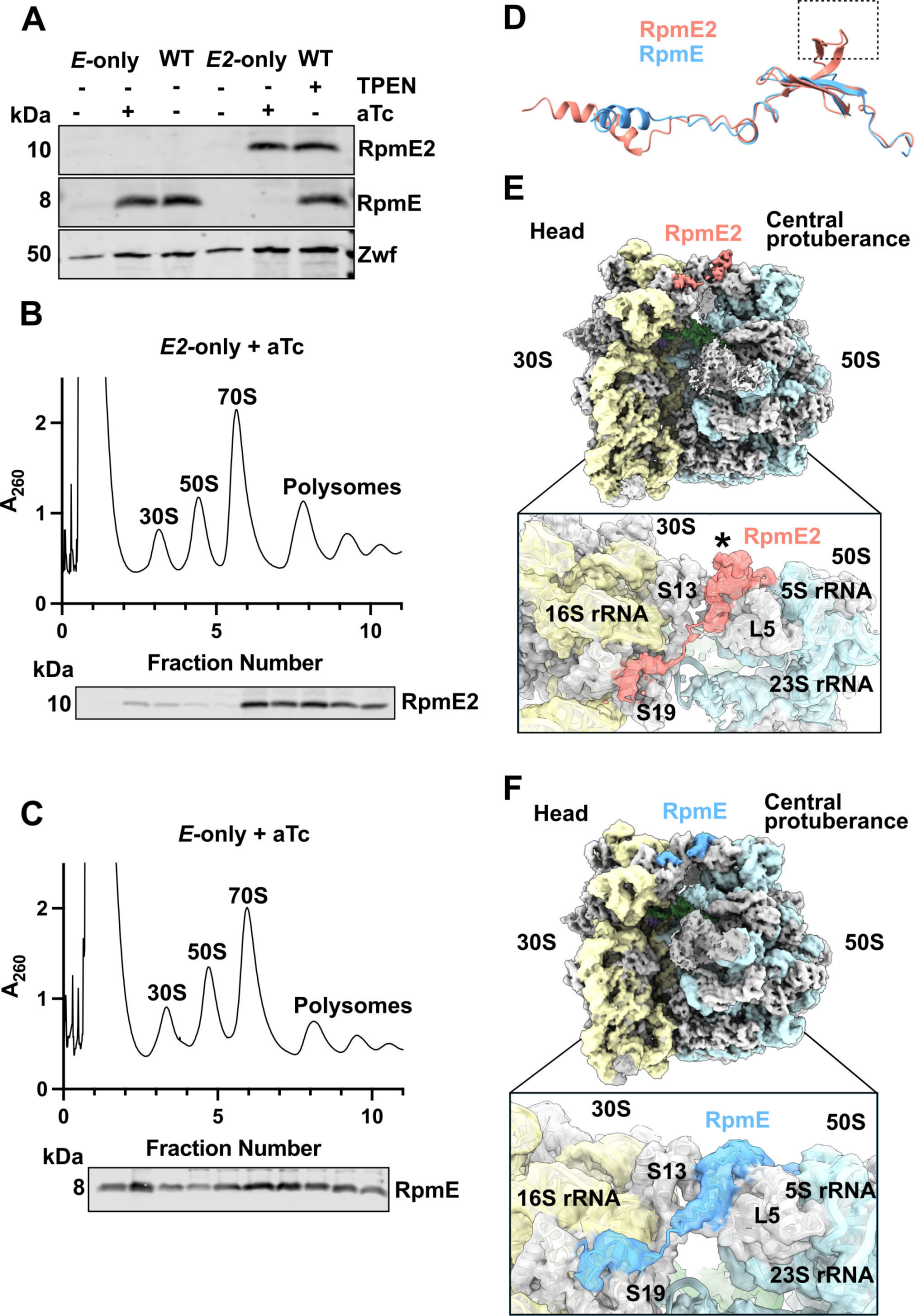
Biochemical and structural features of strains expressing only RpmE or RpmE2. (**A**) WT, Δ*E E*-only, and Δ*E E2*-only were cultured overnight on GCB plates with or without 20 ng/mL aTc and 10 μM TPEN. Whole cell lysates prepared from bacteria in each condition were separated by 4%–16.5% Tris-Tricine SDS-PAGE, transferred to nitrocellulose, and immunoblotted for RpmE2, RpmE, and Zwf (loading control). (**B and C**) Clarified cell extracts from *E2*-only (**B**) or *E*-only (**C**)Gc were applied to a 10%–40% sucrose gradient and subjected to ultracentrifugation, followed by fractionation with UV monitoring. Each trace represents one of three biological replicates. Equal volumes of fractions were TCA-precipitated, resuspended in sample buffer, separated by SDS-PAGE as above, and immunoblotted for RpmE2 or RpmE. Additional traces and immunoblots are shown in Fig. S2. (**D**) AlphaFold-predicted structures of RpmE2 (UniProt: Q5F861) and RpmE (UniProt: Q5F513) were aligned in ChimeraX. The dashed box indicates a loop present in the predicted structure of RpmE2 but not present in RpmE. (**E and F**) Cryo-EM analysis of 70S ribosome-containing fractions from *E2-*only (**E**) and *E-*only (**F**) Gc. Cryo-EM density maps of the Gc translating ribosome are shown. The AlphaFold-predicted structures of RpmE2 (UniProt: Q5F861) or RpmE (UniProt: Q5F513), together with the *E. coli* 70S ribosome model (PDB: 8B0X) (46), were fitted into the respective cryo-EM density maps. The asterisk denotes the “loop” motif in RpmE2. EM densities of the ribosomal proteins are colored gray, rRNA is colored in yellow (small subunit) and teal (large subunit), and tRNA is colored in green. RpmE and RpmE2 are colored blue and orange, respectively.

The enhanced growth of Δ*E2*Δ*J2* Gc in zinc-limited medium led us to hypothesize that RpmE2 and RpmE are not functionally equivalent. To directly test this, Gc strains expressing solely RpmE2 or RpmE were constructed. *rpmE* was insertionally inactivated with a spectinomycin-resistance cassette (Δ*rpmE*) and then complemented at an ectopic location in the chromosome with either the *rpmE* (*E*-only) or *rpmE2* (*E2*-only) ORF, each regulated by an anhydrotetracycline (aTc)-inducible promoter. By immunoblotting, we confirmed each complement only produced its correct L31 protein in an aTc-dependent manner, while the other was not made (Fig. 4A). Under these conditions, native *rpmE2* and *rpmJ2* are not induced (Fig. 1C). When *E2*-only Gc was grown with aTc, RpmE2 was detected in 70S ribosomes and polysomes and less robustly in 50S ribosomal subunits. In contrast, in *E*-only Gc, RpmE was detected in 50S, 70S, and polysome fractions (Fig. 4B and C; Fig. S2). The distribution of RpmE and RpmE2 in these strains mirrors observations for WT Gc grown with and without zinc (Fig. 2F and G; Fig. S1).

AlphaFold-generated structures predicted that Gc RpmE2 contains a loop motif absent from RpmE, as reported in *E. coli* (47) (Fig. 4D). To evaluate how RpmE and RpmE2 might affect ribosome structure, Cryo-EM analysis was performed on *E2*-only and *E*-only 70S ribosomes. AlphaFold predictions of RpmE2 or RpmE structure were fitted to densities on *E2*-only or *E*-only 70S Gc ribosomes, respectively, using the 70S *E. coli* ribosome model (46) (PDB: 8B0X) (Table S2; Fig. S4). Gc RpmE and RpmE2 share sequence similarity with their *E. coli* counterparts (Fig. S3) (42). By this model, RpmE and RpmE2 were predicted to span the 50S and 30S ribosomal subunits, as expected based on the reported position of L31 (38) (Fig. 4E and F). The RpmE2-specific loop motif was represented in the Gc ribosome electron maps (asterisk, Fig. 4E and F). *E2*-only and *E*-only ribosomes also exhibited similar orientation and overall structure (Fig. S5; Movie S1). These findings indicate both L31 proteins are incorporated into Gc ribosomes and indicate structural differences between RpmE- and RpmE2-containing ribosomes.

**Fig 5:**
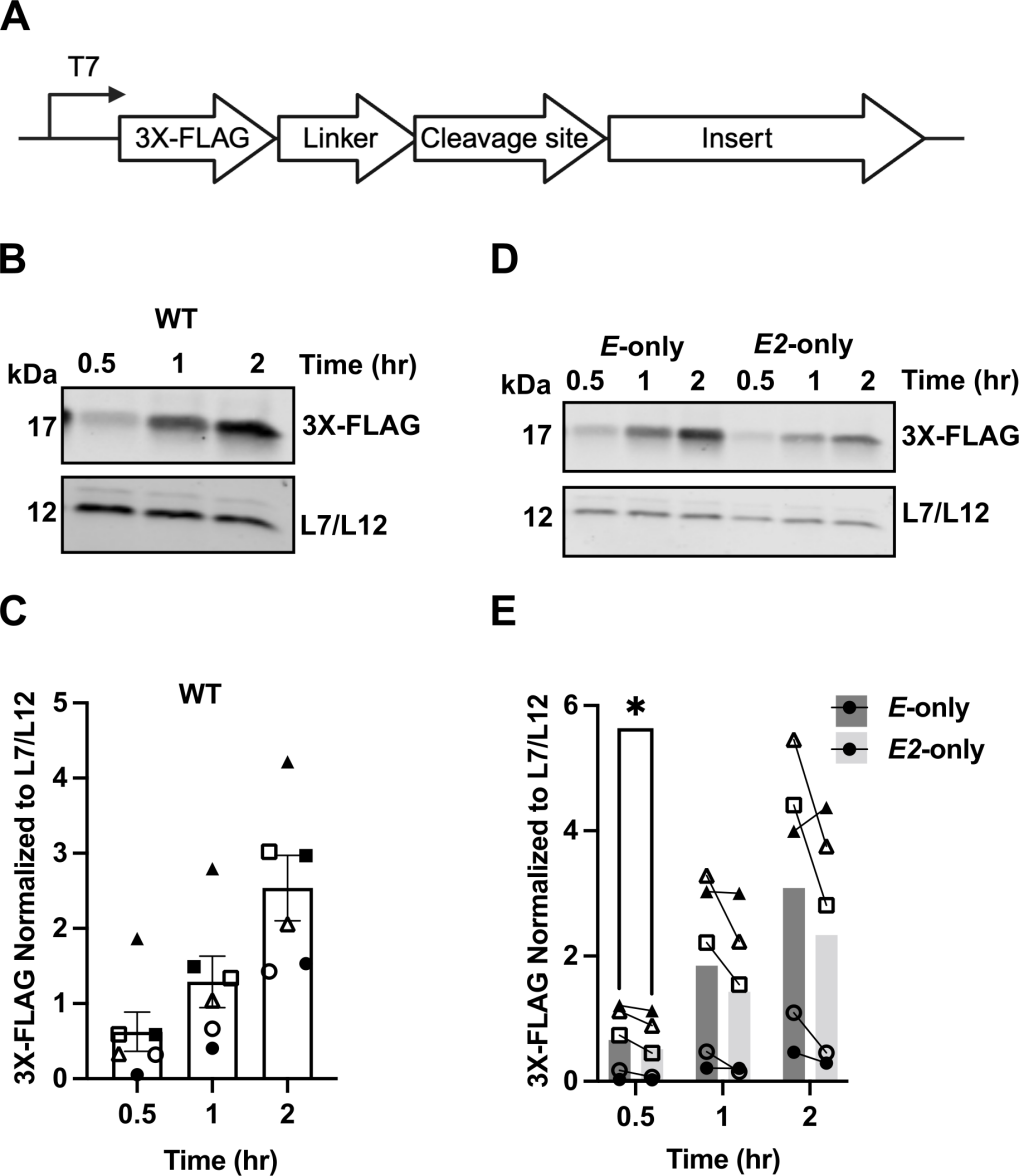
*In vitro* translation characteristics of RpmE-only versus RpmE2-only ribosomes. (**A**) 3×-FLAG-tagged construct used for *in vitro* translation. See Table S1 for sequence. (**B**) Ribosomes were purified by sucrose cushion centrifugation from WT Gc grown to mid-log phase in modified GCBL + 20 ng/mL aTc. Ribosomes (100 nM final) were added to PURexpress *in vitro* translation reactions containing the 3×-FLAG template in panel **A**. Aliquots were removed at 0.5, 1, and 2 h, and protein production was analyzed by 4%–20% Tris-Glycine SDS-PAGE followed by immunoblot for 3×-FLAG and L7/L12 ribosomal protein (loading control). (**C**) Band intensity of 3×-FLAG was analyzed by ImageStudio and normalized to the band intensity of the L7/L12 ribosomal protein. Each symbol represents an independent translation reaction, open/closed shapes represent the ribosome preparation used in the reaction. Data represent the mean ± standard error from the mean. (**D**) Ribosomes were purified by sucrose cushion centrifugation from *E*-only and *E2*-only Gc grown to mid-log phase in modified GCBL + 20 ng/mL aTc and used for *in vitro* translation with a 3×-FLAG encoding template, as in panel **A**. Aliquots were removed at 0.5, 1, and 2 h, and protein production was analyzed as in panel **A**. (**E**) Band intensity of 3X-FLAG was analyzed by ImageStudio and normalized to the band intensity of the L7/L12 ribosomal protein. Each symbol represents an independent translation reaction; open/closed shapes represent the ribosome preparation used in the reaction. Lines match biological replicates. Data in panel **D** represent results from the reactions represented by open triangles. Bars represent the mean, *P*-values were calculated by paired *t*-test for *E*-only versus *E2*-only for each time point: *T* = 0.5 h, *P < 0.05; *T* = 1 h, *P* = 0.106; *T* = 2 h, *P* = 0.137.

To evaluate how RpmE and RpmE2 affect translational capacity of Gc ribosomes, crude ribosomes were isolated from mid-log phase WT Gc, *E*-only, and *E2*-only Gc grown in GCBL containing aTc (zinc-replete conditions). We verified the ribosomes from WT and each aTc-inducible complement contained the correct L31 protein (Fig. S6). Gc ribosomes were used for coupled *in vitro* transcription-translation reactions with a DNA template encoding a non-native protein fused to 3×-FLAG (Fig. 5A) and the non-ribosomal components of the PURexpress *E. coli* system (New England Biosciences). By immunoblot, 3×-FLAG protein abundance increased over time in each reaction, indicating WT, *E*-only, and *E2*-only Gc ribosomes are translationally active *in vitro* (Fig. 5B through E). However, at matched time points, less 3×-FLAG was generated in *E2*-only ribosome reactions relative to *E*-only reactions (Fig. 5D and E). This result suggests that incorporation of RpmE2 trends towards making Gc ribosomes less translationally capable *in vitro*.

**Fig 6:**
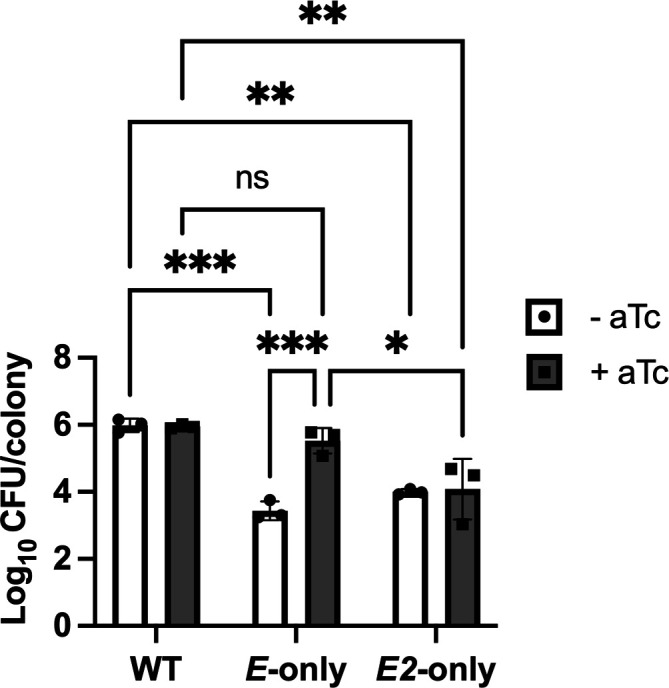
RpmE2-only Gc exhibit a growth defect relative to RpmE-only Gc and WT. WT, Δ*E E*-only, and Δ*E E2*-only Gc were grown for 24 h on GCB plates with or without 20 ng/mL aTc, at which point five single colonies were isolated, resuspended in GCBL, and serially diluted onto GCB or GCB + 20 ng/mL aTc. CFU were enumerated after 24 h, from which CFU per colony was calculated. Data represent the mean of three biological replicates ± standard deviation from the mean. Asterisks represent the results of a two-way ANOVA followed by Tukey’s multiple comparisons test, ***P < 0.001, **P < 0.01, *P < 0.05, ns = not significant.

To assess how RpmE2 and RpmE affect Gc growth independent of zinc, CFU per colony of *E*-only vs *E2*-only Gc was measured. Without aTc, both *E*-only and *E2*-only Gc exhibited a ~2 log reduction in recovered CFU relative to WT (Fig. 6); no L31 protein is produced in these strains without aTc (Fig. 4A). Addition of aTc rescued growth of *E*-only Gc to levels indistinguishable from WT (Fig. 6). In contrast, addition of aTc did not increase growth of *E2*-only Gc, which remained significantly reduced relative to WT or aTc-induced *E*-only Gc (Fig. 6), even though RpmE2 was incorporated into its ribosomes (Fig. 4). We conclude that production of the alternative ribosomal protein RpmE2, which occurs under zinc limitation, reduces translation and decreases growth of Gc.

## DISCUSSION

Gc must adapt to changing zinc availability during pathogenesis. Free zinc is abundant in seminal fluid (12, 13) but limited in the female reproductive tract by abundant calprotectin and psoriasin (11). Gc responds to zinc limitation by derepressing the Zur regulon, which includes outer membrane metal transporters, a periplasmic zinc capture protein, the ZnuABC inner membrane import system, and alternative ribosomal proteins (41, 43). In this study, we uncover the role of an operon encoding two alternative ribosomal proteins, *rpmE2* and *rpmJ2,* in the Gc response to zinc limitation. In contrast to prevailing models of how these proteins contribute to zinc-limited bacterial growth, Gc lacking *rpmE2* and *rpmJ2* grow better, not worse, than WT in zinc-chelated medium. Although RpmE2 is incorporated into ribosomes, it cannot substitute for RpmE in translational capacity, and RpmE2-only bacteria have a growth defect. These results inform a model in which RpmE and RpmE2 are both made and incorporated into ribosomes in zinc-limited Gc, with the RpmE2-containing ribosomes helping to slow translation and growth in bacteria experiencing this adverse condition.

Paralogous ribosomal proteins are encoded in many bacterial species, where the non-zinc-binding alternative protein is produced when zinc is limited. Similarly, we found that *rpmE2* and *rpmJ2* are induced in zinc-limited Gc. However, the functions of Gc RpmE2 and RpmE and their contributions to gonococcal growth are unique and distinct from what has been found in other bacteria. While RpmE2 is produced under zinc limitation and detected in Gc ribosomes, RpmE levels do not change under zinc limitation in Gc, and RpmE is detected in a *zur* mutant in which RpmE2 is overexpressed. It is possible that TPEN treatment to induce zinc limitation has effects on the ribosome or ribosomal protein alternation, which could be investigated in future studies. Overall, we posit that Gc ribosomal protein alternation differs from the model in other bacteria in which the canonical paralog is degraded to liberate zinc under zinc limitation (24, 27).

To our surprise, bacteria lacking RpmE2 and RpmJ2 exhibited a growth advantage in zinc-limited medium. This differs from other bacteria in which deletion of alternative ribosomal proteins confers a growth defect under zinc limitation (23, 24, 27–31). The absence of a growth defect in Gc lacking RpmE2 under zinc limitation, along with retention of RpmE in some ribosomes from zinc-limited Gc, imply that RpmE is not being degraded as a source of zinc, but this remains to be directly tested. We also evaluated the contribution of RpmE2 and RpmJ2 to Gc survival *in vivo* by competitive infection of WT and Δ*E2*Δ*J2* Gc in an established mouse model of Gc colonization (48). Since Gc cannot utilize zinc bound to mouse calprotectin or psoriasin (44, 49), we hypothesized that Gc would be zinc-limited *in vivo*. The average competitive index of WT:Δ*E2*Δ*J2* Gc remained at ~1 for the duration of the experiment (Fig. S7), implying RpmE2 and RpmJ2 are dispensable in this model. However, the mouse genital tract may not have been sufficiently zinc-limited to induce RpmE2 and RpmJ2, so the contribution of alternative ribosomal proteins *in vivo* remains an open question.

Cryo-EM maps of the RpmE2-containing and RpmE-containing ribosomes demonstrated that both proteins span the 50S and 30S subunits, and RpmE2 contains a loop motif absent from RpmE. In *E. coli*, RpmE forms part of a ribosomal intersubunit bridge that limits ratcheting during translation (50), and the loop motif is conserved in RpmE2 yet absent from RpmE (47). The loop in *E. coli* RpmE2 has been proposed to confer increased mobility during ribosomal ratcheting (47), which could lead to frameshifting and translational dysregulation. The surface exposure of the RpmE2 loop also suggests that it could be targeted for proteolytic processing, as a means of ribosome regulation when zinc is limited. Structural evaluation of translating RpmE and RpmE2 Gc ribosomes will reveal the role of the loop motif and other features of Gc RpmE2. Intriguingly, polysome profiling of RpmE2- versus RpmE-producing Gc strains suggests that RpmE2 may not be present in 50S ribosomes, whereas RpmE is. While further analysis is necessary to confirm this, such a result could indicate differences in ribosome occupancy or splitting between RpmE2- and RpmE-containing ribosomes. In *E. coli,* absence of both L31 ribosomal proteins decreases translation, increases frameshifting, and reduces ribosome processivity (21, 33). If RpmE2-only Gc contains more ribosomes with an empty L31 site, this could explain its reduced *in vitro* translation efficiency, a possibility for future study.

Our findings indicate that Gc RpmE2 is not equivalent to RpmE in translation. Isolated RpmE2-containing ribosomes exhibited an *in vitro* protein synthesis defect relative to RpmE-containing ribosomes. This differs from *E. coli*, in which RpmE2 and RpmE, and RpmJ2 and RpmJ, exhibit similar translation *in vitro* (21). One caveat to our results is that *E. coli* translation factors were used for *in vitro* translation; RpmE2-containing ribosomes may not work as efficiently with non-*Neisseria* factors or the transcript template encoding a non-native FLAG-containing peptide. Moreover, we did not examine RpmJ2 versus RpmJ in Gc translation. Since the L36 protein is buried deeper in the ribosome and interacts with 23S rRNA, if RpmJ and RpmJ2 are not equivalent, RpmJ2 could amplify the effect of RpmE2 on Gc translation. The effect of L31 and L36 alternation on ribosomal translation of native transcripts, processivity, and frameshifting in intact Gc are subjects for future work.

We found it striking that RpmE2-only Gc have a statistically significant growth defect relative to WT and RpmE-only bacteria, further evidence that RpmE and RpmE2 are not functionally equivalent in Gc. In *E. coli,* RpmE2-only bacteria exhibit a modest growth defect relative to WT at 37°C (33). A hypothesis for future testing is that the growth defect of RpmE2-only Gc is driven by its reduced translational capacity. It is also possible that zinc-limited Gc produce additional ribosome-associated factors that modulate functionality of RpmE2-containing ribosomes, but these factors would not be made in the zinc-replete conditions used for the RpmE2-only studies. Notably, at 8 h, the viability of zinc-limited WT Gc decreased compared to 6 h; whether bacteria are dying at this time point due to reduced translation will require evaluation of single bacteria cultured in these different conditions. Additional growth and ribosomal structure and function studies are needed to understand how RpmE2 affects Gc growth and should also be conducted with Gc that produce both RpmJ2 and RpmE2 but not their canonical counterparts, which is physiologically relevant given they are coordinately induced in zinc-limited Gc.

Zinc acquisition is essential for Gc pathogenesis, and zinc acquisition factors are potential vaccine targets to “starve and kill” the pathogen (51). Our data suggest that Gc also induces alternative ribosomal protein RpmE2 to adapt to zinc limitation. Ribosomal protein alternation in zinc-limited Gc represents a new mechanism by which this pathogen has adapted to its host environment that could broadly impact pathogenesis. Reduced translational efficacy conferred by RpmE2 could also enhance resistance to ribosome-targeting antibiotics, such as aminoglycosides, which are less effective during ribosome hibernation (52). Increased frameshifting conferred by RpmE2, as in *E. coli* (33), could increase Gc phase variation, resulting in progeny that have a survival advantage during infection. Overall, we hypothesize RpmE2 production in Gc reduces translation to slow bacterial growth under zinc-limited conditions, enabling Gc to adapt to adverse growth conditions such as those encountered during mucosal infection in its obligate human host.

## MATERIALS AND METHODS

### Bacterial strain construction

Wild type (WT) in this study is piliated Opaless FA1090 (53), except where otherwise noted. FA1090 Δ*zur* was previously described (54). Δ*rpmE2*Δ*rpmJ2* and Δ*rpmE* strains were generated by spot transformation of WT Gc with linear PCR constructs encoding kanamycin or spectinomycin resistance cassettes disrupting the ORFs, respectively, using standard methods (55). Δ*rpmE2*Δ*rpmJ2* and Δ*rpmE* transformants were selected on gonococcal base agar (GCB) plates supplemented with 40 μg/mL kanamycin or 80 μg/mL spectinomycin and 10 μM TPEN, respectively, as described in reference 55. To generate *E*-only and *E2*-only strains, *rpmE* and *rpmE2* ORFs were cloned into the pMR68 vector (56) and plasmid DNA was used to spot transform Δ*rpmE*. Transformants were selected on GCB supplemented with 0.3 μg/mL erythromycin. To make Δ*rpmE2*Δ*rpmJ2* in the constitutively producing OpaD FA1090 background (53), genomic DNA from Δ*rpmE2*Δ*rpmJ2* in the Opaless background was used as a template for backcrossing into OpaD, with antibiotic selection. Retention of OpaD was confirmed by colony opacity. See supplemental methods for detailed procedures.

### Bacterial growth conditions

Unless otherwise noted, Gc was inoculated from −80°C onto GCB agar plates and incubated at 37°C, 5% CO_2_ for 16 h. Lawns were made from single colonies onto GCB agar plates and incubated at 37°C, 5% CO_2_ for 8 h. Bacteria were inoculated into GCBL with Kellogg’s supplements (modified GCBL) (57), incubated with rotation overnight at 30°C, and grown at 37°C prior to use. The *E*-only and *E2*-only strains were cultured for 24 h for single colonies and 24–32 h for lawns on GCB to account for the growth defect in these strains without aTc. *E. coli* was inoculated from −80°C onto LB agar plates with appropriate antibiotics and incubated overnight at 37°C. Single colonies were inoculated from plates into LB medium with antibiotic selection and cultures were grown rotating at 37°C.

### RNA extraction, RT-PCR, and qRT-PCR

Overnight cultures of WT, FA1090, and FA1090 Δ*zur* Gc in modified GCBL were back-diluted twice prior to inoculation in modified GCBL with or without TPEN (20 µM) and ZnSO_4_ (10 µM). Gc were grown to mid-log phase (*T* = 3 h), normalized to OD = 0.16 (1 × 10^8^ CFU), and preserved in RNAlater (Ambion) with storage at −80°C. Samples were treated with EDTA, lysozyme, and proteinase K for lysis and digestion prior to RNA extraction with the Qiagen RNeasy or RNeasy Plus Mini kits. A total of 50 ng of RNA was subjected to qRT-PCR with Power SYBR Green Master Mix (Thermo Fisher Scientific), gene-specific primers, and comparative Ct analysis. Data were normalized to *ngo1823* (L15 ribosomal protein) (Fig. 1C) or 5S rRNA (Fig. 1D) and WT Gc in untreated medium (FA1090 for Fig. 1D). For RT-PCR, DNase-treated RNA from Gc treated with 20 µM TPEN was subjected to reverse transcription with the High Capacity cDNA Reverse Transcription Kit (Thermo Fisher Scientific), followed by PCR with primers spanning *rpmE2-rpmJ2* or *rpmE2* and *rpmJ2* alone (Table S1). PCR products were subject to electrophoresis on a 2% agarose gel followed by UV exposure and imaging via Chemidoc (Bio Rad). Reverse transcription reactions with no reverse transcriptase or no template served as negative controls.

### Lysate generation and SDS-PAGE

Single colonies of WT Gc were inoculated onto GCB plates and cultured overnight at 37°C, 5% CO_2._ Lawns were inoculated into modified GCBL with or without TPEN (20 µM) or ZnSO_4_ (10 µM). At 3, 6, 9, and 24 h post-inoculation, 10^8^ CFU were collected and pelleted, and remaining culture was diluted in fresh media to OD_550_ = 0.16. Pellets were resuspended in 1× Tris-tricine sample buffer (50 mM Tris-HCl [pH 6.8], 10% glycerol, 2% SDS, 2% β-mercaptoethanol, 0.025% bromophenol blue) or 1× BioRad Tris-tricine sample buffer (100 mM Tris-Cl, pH 6.8, 20% glycerol, 1% SDS, 1% β-mercaptoethanol, 0.02% Coomassie blue) and boiled for 5 min. Lysates were resolved by 16.5% Tris-tricine SDS-PAGE and transferred to a 0.2-µm nitrocellulose membrane for immunoblotting with RpmE, RpmE2, Zwf, or L7/L12 specific antisera. See supplemental materials for details on immunoblotting conditions.

To evaluate RpmE2 and RpmE production in Δ*zur*, lysates were similarly prepared from 10^8^ CFU of log-phase (*T* = 6 h) Δ*zur* Gc grown in modified GCBL with TPEN (20 µM) and with or without zinc (10 µM).

To confirm aTc-dependent induction of RpmE2 and RpmE in complemented strains, WT, *E*-only, and *E2*-only Gc were inoculated from single colonies onto GCB plates with or without 10 μM TPEN and 20 ng/mL aTc and grown overnight at 37°C, 5% CO_2_, and lysates were prepared as above.

### Polysome profiling

Clarified lysates from mid-log phase (OD = 0.4–0.7) WT, *E*-only, or *E2*-only Gc with or without TPEN (20–25 µM) and ZnSO_4_ (10 µM) or aTc (20 ng/mL, for inducible complement strains only) were overlaid on a 10%–40% sucrose gradient and subjected to ultracentrifugation using a Beckman SW41 rotor at 36,000 rpm for 2.5 h at 4°C. Sucrose gradients were fractionated by BioComp gradient fractionator (BioComp Instruments) and the *A*_260_ monitored. Fractions were precipitated with 80% trichloroacetic acid and resuspended in 2× Tris-tricine sample buffer or 2× BioRad Tris-tricine sample buffer prior to SDS-PAGE. See supplemental methods for additional details.

### Gc growth assays

#### Liquid growth in CDM

Nonpiliated OpaD Gc and OpaDΔ*E2J2* Gc were struck from −80°C for single colonies onto GCB plates and incubated overnight at 37°C, 5% CO_2_. Single colonies were resuspended into 1× Chelex-treated chemically defined medium (CDM) (58) to an OD_550_ = 0.05 and incubated for 2 h at 37°C. Gc were diluted to 2 × 10^6^ CFU/mL in CDM with 1 μM Fe(NO_3_)_3_, which supports growth in this medium (59), and with or without 0.25 μM TPEN and 2.5 μM ZnSO_4_. At 0, 6, and 8 h post-inoculation, 20 μL was removed from each condition for serial dilution and plating on GCB to enumerate CFU/mL.

#### CFU per colony

WT, *E*-only, and *E2*-only Gc were struck from frozen at −80°C onto GCB plates with or without 20 ng/mL aTc and incubated at 37°C, 5% CO_2_ for 24 h. Five piliated colonies were swabbed up from plates, resuspended in GCBL, serially diluted, and plated on GCB with or without aTc and incubated at 37°C, 5% CO_2_. The next day, CFU were enumerated and divided by five to yield CFU/colony.

### Gc infection of mice

The female murine model of gonorrhea (48) was performed in accordance with protocol #4153, approved by the University of Virginia Institutional Animal Care and Use Committee. On day 0, 4–6-week-old BALB/c female mice (Charles River Laboratories) were inoculated vaginally with 10^6^ CFU of 1:1 WT (OpaD) and OpaDΔ*E2*Δ*J2* Gc (~5 × 10^5^ CFU/strain). Mice were infected in two groups on two separate days, for group 1 *N* = 10 mice, for group 2 *N* = 4 mice. At days 1, 3, and 5 post-infection mice were swabbed vaginally, and swabs were suspended in 1× PBS that was serially diluted to enumerate CFU. Dilutions were plated on GCB + 100 µg/mL streptomycin to select for Gc, and GCB + 100 µg/mL streptomycin + 50 µg/mL kanamycin to calculate CFU of kanamycin-resistant Δ*E2*Δ*J2* Gc. WT Gc CFU was calculated as total Gc CFU minus Δ*E2*Δ*J2* Gc CFU. Competitive index (CI) was calculated as the ratio of Δ*E2*Δ*J2* CFU to WT CFU in the output to the ratio of Δ*E2*Δ*J2* CFU to WT CFU in the input for each mouse, each day post-infection. See supplemental methods for additional details.

### Predicted structures of RpmE2 and RpmE

RpmE and RpmE2 models were generated in AlphaFold 3 (https://alphafoldserver.com/, accessed 01-09-2026) from strain FA1090 amino acid sequences (NCBI reference sequence NC_002946.2) (60). Models were visualized and aligned in UCSF ChimeraX (v. 1.11rc202512130101) (61).

### Electron microscopy grid preparation and image processing

70S ribosome fractions from sucrose gradients were pooled and concentrated using a 100-kDa MWCO Amicon Ultra-4 centrifugal filter. Sucrose buffer was exchanged four times with buffer containing 10 mM Tris-HCl (pH 7.5), 50 mM KCl, 10 mM MgCl_2_, and 6 mM β-mercaptoethanol. Samples were concentrated by repeated centrifugation at 4,500 × *g* for 5 min at 4°C. Concentrated samples, at 250 ng/μL, were used for cryo-EM grid preparation. Quantifoil R2/1 300-mesh copper grids (Quantifoil, Q350CR1) coated with a 3.6-nm continuous carbon were subjected to glow discharge at 15 mA for 15 s using an Electron Microscopy Sciences glow discharger. Five microliters of reaction mixture was applied to the grid and incubated for 1 min at 4°C in 100% humidity. Grids were rapidly plunged into pre-cooled liquid ethane using ThermoFisher Vitrobot. Data collections were performed at 200 kV with a Falcon4 direct electron detector using a Thermo Fisher Scientific Glacios transmission electron microscope. Images were acquired at 150,000× magnification, yielding a calibrated pixel size of 0.92 Å. The defocus range was maintained between −2.4 and −0.4 µm and a step size of 0.2 µm. See supplemental materials for details on image processing.

RpmE2 (UniProt: Q5F861) and RpmE (UniProt: Q5F513) predicted structures used for modeling were generated in AlphaFold 3 (https://alphafoldserver.com/, accessed 8 October 2024) (60).

### *In vitro* translation

WT, *E*-only, and *E2*-only Gc were grown to mid-log phase in modified GCBL + 20 ng/mL aTc, and ribosomes were isolated for *in vitro* translation as described in reference (62). Clarified lysates were overlaid on a 37.7% sucrose cushion (20 mM Tris-acetate [pH 7.4], 100 mM ammonium chloride, 10 mM magnesium acetate, 0.5 mM EDTA, 6 mM β-mercaptoethanol, and 37.7% sucrose) and spun in a TLA100.3 rotor at 85,000 rpm for 2 h at 4°C. Ribosomes were resuspended in ribosome gradient buffer (20 mM Tris-acetate [pH 7.4, 4°C], 60 mM ammonium chloride, 7.5 mM magnesium acetate, 6 mM β-mercaptoethanol, and 0.5 mM EDTA), and the *A*_260_ value was measured. Ribosomes were diluted to 100 nM and added to the PURexpress Δ Ribosome Kit (New England Biosciences). A gBlock encoding a protein product with a 3X-FLAG tag (Integrated DNA Technologies) was amplified by PCR (Table S1), and 25 ng of gBlock or PCR amplicon was added to each reaction. Reactions were incubated at 37°C, and aliquots were removed at 0.5, 1, and 2 h, diluted in 2.5× sample buffer (30 mM Tris pH 6.8, 12.5% glycerol, 1% SDS, 2.5% β-mercaptoethanol, 0.05% bromophenol blue), and boiled for 2–3 min. Then, 15 μL of sample was resolved by 4%–20% Tris-glycine SDS-PAGE, followed by transfer to a nitrocellulose membrane. See supplemental methods for details on immunoblotting.

For detection of RpmE2 and RpmE in ribosome preparations, WT, *E*-only, and *E2*-only ribosomes were diluted to 17 nM in 1× BioRad Tris-tricine sample buffer and boiled for 2–3 min. Samples were resolved by 16.5% Tris-Tricine SDS-PAGE, transferred to 0.2-µm nitrocellulose, and immunoblotted for RpmE2, RpmE, and L7/L12.

### Figure generation and statistical analyses

Graphs and statistical analyses were generated in GraphPad Prism 10.1.1 for macOS, GraphPad Software, Boston, MA, USA (https://www.graphpad.com/). Statistical tests are described for each figure.

## Data Availability

Cryo-EM maps have been deposited at the Electron Microscopy Data Bank under accession codes EMD-76892 (70S-RpmE) and EMD-76891 (70S-RpmE2).

## SUPPLEMENTAL MATERIAL

Supplemental Material mbio.01137-26-s0001.pdf Supplemental figures, tables, methods, and references.

Movie S1 mbio.01137-26-s0002.mp4 Rotated overlay of density maps from RpmE-only and RpmE2-only ribosomes.

ASM does not own the copyrights to Supplemental Material that may be linked to, or accessed through, an article. The authors have granted ASM a non-exclusive, world-wide license to publish the Supplemental Material files. Please contact the corresponding author directly for reuse.
