# Supplementary material for: Alternative ribosomal protein RpmE2 is produced under zinc limitation in Neisseria gonorrhoeae and slows translation and bacterial growth
Source: mBio. 2026 Jun 18;17(7):e01137-26. doi: 10.1128/mbio.01137-26 (PMC13343924; doi:10.1128/mbio.01137-26)
Supplement: Supplemental Material — Supplemental figures, tables, methods, and references. [file mbio.01137-26-s0001.pdf]

Supplementary information for

Alternative ribosomal protein RpmE2 is produced under zinc limitation in *Neisseria gonorrhoeae* and slows translation and bacterial growth

Amy L. Forehand, Kinga Malezyna, Keena S. Thomas, Ian J. Glomski, Cynthia Nau Cornelissen, Ahmad Jomaa, Alison K. Criss

Supplementary Methods

Bacterial strain construction

To generate the Δ*rpmE2*Δ*rpmJ2* linear construct, three PCR fragments corresponding to ~500 bp upstream and downstream of the *rpmE2rpmJ2* operon and a kanamycin cassette from FA1090 Δ*ngo554*(1) were PCR amplified and then combined into a single amplicon by overlap PCR (see Table S1 for primer sequences). Δ*rpmE* was generated by spot transformation of WT Gc with the linear Δ*rpmE* PCR construct. Transformants were selected on GCB plates supplemented with 80 μg/mL spectinomycin and 10 μM TPEN. Correct insertion of the spectinomycin cassette in the Gc genome was confirmed by PCR and whole genome sequencing (Plasmidsaurus).

To construct Δ*rpmE,* the Ω cassette from FA6815(2) was amplified by PCR along with ~1000 bp upstream and downstream of, and partially including, the *rpmE* ORF (see Table 1 for primer sequences). To generate *rpmE* and *rpmE2* complements, the *rpmE* and *rpmE2* ORFs were amplified with PCR primers containing SacI (forward) and EcoRV (reverse) cut sites (Table S1), digested with SacI and EcoRV enzymes, and inserted into the pMR68 vector(3). pMR68-*rpmE* and pMR68-*rpmE2* were transformed into TOP10 *E. coli* with selection on lysogeny broth agar plates (LB) with 300 μg/mL erythromycin. Proper insertion of *rpmE* and *rpmE2* was confirmed by PCR and Sanger sequencing (Eton Biosciences).

To generate Δ*E E*-only and Δ*E E2*-only, pMR68-*rpmE* and pMR68-*rpmE2* plasmid DNA was spot transformed into Δ*rpmE* and transformants were selected on GCB supplemented with 0.3 μg/mL erythromycin. Δ*E E*-only and Δ*E E2*-only were verified by

PCR and Sanger sequencing (Eton Biosciences) or whole genome sequencing (Plasmidsaurus).

Immunoblotting

Detection of RpmE, RpmE2, and Zwf

Membranes were blocked in 2.5% bovine serum albumin in 1X TBST (0.5 M Tris, 151 mM NaCl, pH 7.6, 0.2% Tween-20) and probed with affinity-purified chicken anti-RpmE or guinea pig anti-RpmE2 (Biosynth). As a loading control, membranes were blocked in 5% bovine serum albumin in 1X TBS-T and probed with rabbit anti-Zwf antisera (loading control(4)). Membranes were stained with fluorescently conjugated anti-rabbit, anti-guinea pig, and anti-chicken antibodies for visualization with LI-COR Odyssey CLx and quantification in Image Studio (5.2).

Detection of 3X-FLAG and L7/L12

Membranes were blocked in 5% nonfat dry milk in 1X TBS-T and probed with monoclonal mouse anti-FLAG M2 antibody (Sigma) and rabbit anti-*S. aureus* 50S L7/L12 (Abcam), followed by fluorescently conjugated goat anti-mouse and goat anti-rabbit antibodies. For detection of L7/L12 in *in vitro* translation ribosome preparations, membranes were blocked in 2.5% bovine serum albumin in 1X TBS-T prior to immunoblotting. Blots were imaged using LI-COR Odyssey CLx and bands were quantified in Image Studio (5.2). Adjustments to image display (signal, background, midtones) were done in Image Studio (5.2) and applied to the entire image. These adjustments did not alter the underlying image data for quantification.

Bacterial lysis for polysome profiling and in vitro translation

Polysome profiling

Overnight cultures of WT, *E*-only, or *E2*-only Gc with or without TPEN (20-25 μM) and $ZnSO_4$ (10 μM) or aTc (20 ng/mL, for inducible complement strains only) were cultured at 37°C, 5% CO_2 with shaking until mid-log phase (OD=0.5-0.7) and prepared for polysome profiling using a protocol adapted from (5). TPEN concentrations are listed for each polysome profile and were empirically determined to induce RpmE2 production. Chloramphenicol was added to 0.1 mg/mL for 2-3 min before chilling cultures on ice and pelleting bacteria. Pellets were resuspended in 20 mM Tris-HCl, pH 7.5, 15 mM $MgCl_2$ (lysis buffer), frozen in liquid nitrogen, and stored at -80°C. Prior to polysome profiling, lysozyme was added to 1 mg/mL and bacteria were lysed by three freeze-thaw cycles in liquid nitrogen and a 30° water bath and treated with 0.3% sodium deoxycholate and 1.8 μg/mL DNase I. To remove cellular debris and membranes, lysates were centrifuged at 14,000xg for 20 min and 14,000xg for 10 min. Supernatant was collected and normalized to A_{260}=17 in lysis buffer.

In vitro translation

WT, *E*-only, and *E2*-only Gc were cultured to mid-log phase in modified GCBL + 20 ng/mL aTc, cooled on ice and pelleted at 8,500xg for 15 minutes at 4°C(6). Cells were resuspended in ribosome gradient buffer (20 mM Tris-acetate [pH 7.4, 4°C], 60 mM ammonium chloride, 7.5 mM magnesium acetate, 6 mM β-mercaptoethanol, and 0.5 mM EDTA)(7) and frozen in liquid nitrogen. Bacteria were lysed by three freeze-thaw cycles in liquid nitrogen and a 30 °C water bath and treated with sodium deoxycholate and

DNase I, as described for polysome profiling. Lysate was cleared of debris by centrifugation at 17,000 xg for 25 minutes at 4°C.

Amino acid sequence alignments

Gc strain FA1090 (NCBI reference sequence NC_002946.2) amino acid sequences were used for Gc RpmE, RpmE2, RpmJ, and RpmJ2. Amino acid sequences from *E. coli* strain BL21 were used for *E. coli* RpmE and RpmE2(8). Sequences were aligned in UniProt(9).

Gc mouse model

4-6 week old BALB/c female mice (Charles River Laboratories) were maintained in single-sex housing with autoclaved food and drinking water. Mice were treated with 0.1 mL of 5 mg/mL β-estradiol (Sigma) subcutaneously on days -2, 0, and 2 to synchronize the estrous cycle to increase susceptibility to Gc infection(10). Mice received 0.2 mL of 24 mg/mL streptomycin (X-gen) and 4 mg/mL vancomycin (Hikma) intraperitoneally on days -2 and -1, and 0.15 mL of 24 mg/mL streptomycin and 4 mg/mL vancomycin intraperitoneally on day 0 and day 1. On day -2, 0.4 g/L trimethoprim sulfate (Sigma) was added to the drinking water and was replaced with fresh treated water on days 0, 2, and 4. Starting on day 2, 5g/L streptomycin sulfate (Sigma) was added to the drinking water and was replaced with fresh treated water at day 4. These antibiotic treatments are administered to prevent outgrowth of commensal flora, as previously described (10).

Cryo-EM Image Processing and Map Alignment

2,298 micrographs were collected for ribosomes containing RpmE2, and 3,006 micrographs were collected for ribosomes containing RpmE. Motion correction, dose

weighting, and contrast transfer function (CTF) estimation were performed in CryoSPARC (11). Particles were picked using the blob picker with the diameter range (150–350 Å), yielding 627,175 particles extracted with a box size of 512 × 512 pixels for RpmE2-containing ribosomes and 477,888 particles extracted with a box size of 450 × 450 pixels for RpmE-containing ribosomes. RpmE2-containing ribosome particles were classified into 50 2D classes, of which 26 classes were selected. RpmE-containing ribosome particles were classified into 100 2D classes, from which 25 classes were retained for further analysis. The RpmE2-containing ribosome retained particles were used to generate an ab initio reconstruction, followed by two rounds of heterogeneous refinement. RpmE-containing ribosome particles were used for two rounds of heterogeneous refinement, with the volume generated from homogeneous refinement of the 70S ribosome dataset containing RpmE2 as a reference. For RpmE2-containing ribosomes, 95,434 particles were refined using homogeneous refinement, producing a reconstruction of the 70S ribosome with resolved RpmE2 density. The final map achieved a global resolution of 3.27 Å, as estimated by Fourier shell correlation (FSC) at the 0.143 criterion, and was low-pass filtered to 3.5 Å for visualization. The RpmE 70S ribosome dataset, containing a total of 81,249 particles, was refined using homogeneous refinement, followed by non-uniform refinement that resulted in a consensus reconstruction of the 70S ribosome with density corresponding to RpmE. The final map converged to a global resolution of 3.21 Å, as determined by FSC at the 0.143 criterion, and was low-pass filtered to 3.5 Å for visualization. Cryo-EM map alignment and movie generation were performed using UCSF ChimeraX (v1.8)(12).

Table S1. Primers and gBlocks used in this study.

Primer	Sequence	Application
Δ*rpmE2*Δ*rpmJ2* 1 Fwd	GGTACCGCGATTTGGCGCAATTCGTCG	Overlap PCR
Δ*rpmE2*Δ*rpmJ2* 1 Rev	GGCTCATAACACCCCTTACTTTATCATAATTTAGAAGGC	Overlap PCR
Δ*rpmE2*Δ*rpmJ2* 2 Fwd	CTTCTAAATTATGATAAAGTAAGGGGTGTTATGAGCCATATTCAACG	Overlap PCR
Δ*rpmE2*Δ*rpmJ2* 2 Rev	GGTTCAACTCAGCAAAAGTTCGATTTATTCAACAAAGC	Overlap PCR
Δ*rpmE2*Δ*rpmJ2* 3 Fwd	CTGAGTTGAACCCCTGCCCTTCC	Overlap PCR
Δ*rpmE2*Δ*rpmJ2* 3 Rev	GCGGCCGCGCGTTACAATGCCCACCGAGG	Overlap PCR
Δ*rpmE* 1 Fwd	GGTACCACGTTCAGACGGCATTCAACC	Overlap PCR
Δ*rpmE* 1 Rev	GGGGAATTGATCCCAAACCTCGATGTTAAAGTTTTC	Overlap PCR
Δ*rpmE* 2 Fwd	ATCGAGGTTTGGGATCAATTCCCCTG	Overlap PCR
Δ*rpmE* 2 Rev	GTATAGAACGGGATCAGCTTAGTAAAGCC	Overlap PCR
Δ*rpmE* 3 Fwd	GGGCTTTACTAAGCTGATCCCGTTCTATACCG	Overlap PCR
Δ*rpmE* 3 Rev	CCGCGGAAGCGTGAGAAGAAATGGAC	Overlap PCR
rpmE SacI Fwd	TCAGAGCTCGGTACATCCCCTATG	Cloning
rpmE EcoRV Rev	GATATCATGGCAGGGCTTAATTCTTGC	Cloning
rpmE2 SacI Fwd	GAGCTCCTTCAAGCAAGGCAATATATGAAACC	Cloning
rpmE2 EcoRV Rev	GATATCGAGCGAAGACAGAACCTGC	Cloning
ngo1823 Fwd	CCCTCGAAACCCACCTTATG	qRT-PCR
ngo1823 Rev	CGGTATTGGTAGTGGTCTTGG	qRT-PCR
ngo1049 Fwd	ACTCGAACCCATCGCCAAAG	qRT-PCR
ngo1049 Rev	GGTAACGAGGTAGCTGCCGT	qRT-PCR
rpmE2 Fwd	AATGAAGGCTGGCTGATTCG	qRT-PCR, RT-PCR (primer #2)[1]
rpmE2 Rev	ATAGACGGGATGTGAGGAAGAG	qRT-PCR
rpmE Fwd	GAAACAAGGTATTCACCCGAAC	qRT-PCR
rpmE Rev	GGAGCAAACCTCGATGTTAAAG	qRT-PCR
rpmJ2 Fwd	CAGGTTCTGTCTTCGCTCAA	qRT-PCR, RT-PCR (primer #4)
rpmJ2 Rev	CAGCGTTGGCGCGAT	qRT-PCR, RT-PCR (primer #5)
rpmJ Fwd	GCGTGTACAACCATCTGTTAAG	qRT-PCR

rpmJ Rev	CAGTACAAATCACACGAACTACAC	qRT-PCR
5S rRNA Fwd	CGGCCATAGCGAGTTGGT	qRT-PCR
5S rRNA Rev	TTGGCAGTGACCTACTTTCG	qRT-PCR
rpmE2 Fwd	CAGGTTCTGTCTTCGCTCAA	RT-PCR(primer #1)
rpmE2 Rev	CAGGTTCTGTCTTCGCTCAA	RT-PCR(primer #3)
FLAG gBlock Fwd	GCTCTTCTAAAGACCTTACCC	gBlock amplification
FLAG gBlock Rev	GCGAGTTCTGGCTGGCTTG	gBlock amplification
3X-FLAG gBlock	GCTCTTCTAAAGACCTTACCCTACTTCTAATTCTAATACGACTCACTATAGGGAACAACAACAACCGTTAGCTTCCGACACAAGGCTTTTCACTAGCAACTAAGGAGGTCCACCATGGCCTACAAAGACCACGACGGTGATTATAAAGATCACGACATCGATTACAAGGACGACGACGACAAGCTGCCGGATAACCACTATCTGAGCACCCAGAGCGCGCTGAGCAAGGACCCGAACGAGAAACCCACTACTCTGGAGGTCTTATTTCAAGGCCCAGGTGGCTCAATACGTGATCACATGGTGCTGCTGGAGTTCGTTGATCACCACCTCGGGATCAGCGGCGGAAAGAAAATTACAAACGTGGATATGGCCCGGTATATGGCACAGACAAACACTCGGCGTGGTGTGACTAGCAACATGAAATATAAAAACGTTGCCATCATGACTGATGCGGACCCAGACTAGCGGGGTTAATCGACTGGTGGGGTTCAATTTACCCATGTCTACTCGCGTTCTTTAGTCGTTGGCCAGAACTGTACAACCGGGGCTGCGTAGTCACGCTGAAAGATTCTAAAGGAAAGATCAAGAATTCCCAACTGCACGTGCAGGTCGACGGTGTTTTTGCAGGACATCATCACCACCATCATTAGTGCGGCCGCACAGCTGTATACACGTGCAAGCCAGCCAGAACTCGC	*In vitro* translation

[1] Numbers after RT-PCR primers refer to the schematic in Fig. 1D.

Table S2: Cryo-EM data collection.

Data collection and processing	70S containing RpmE2	70S containing RpmE
Nominal magnification	150,000	150,000
Voltage (kV)	200 kV	200 kV
Electron dose ($e^-/Å^2$)	40	40
Defocus range (μm)	-2.4 to -0.4 μm	-2.4 to -0.4 μm
Pixel size (Å)	0.92	0.92
Total micrographs (no.)	2,298	3,006
Initial particle images (no.)	627,175	477,888
Final particle images (no.)	95,434	81,249
Map resolution at FSC = 0.143 (Å)	3.27	3.21

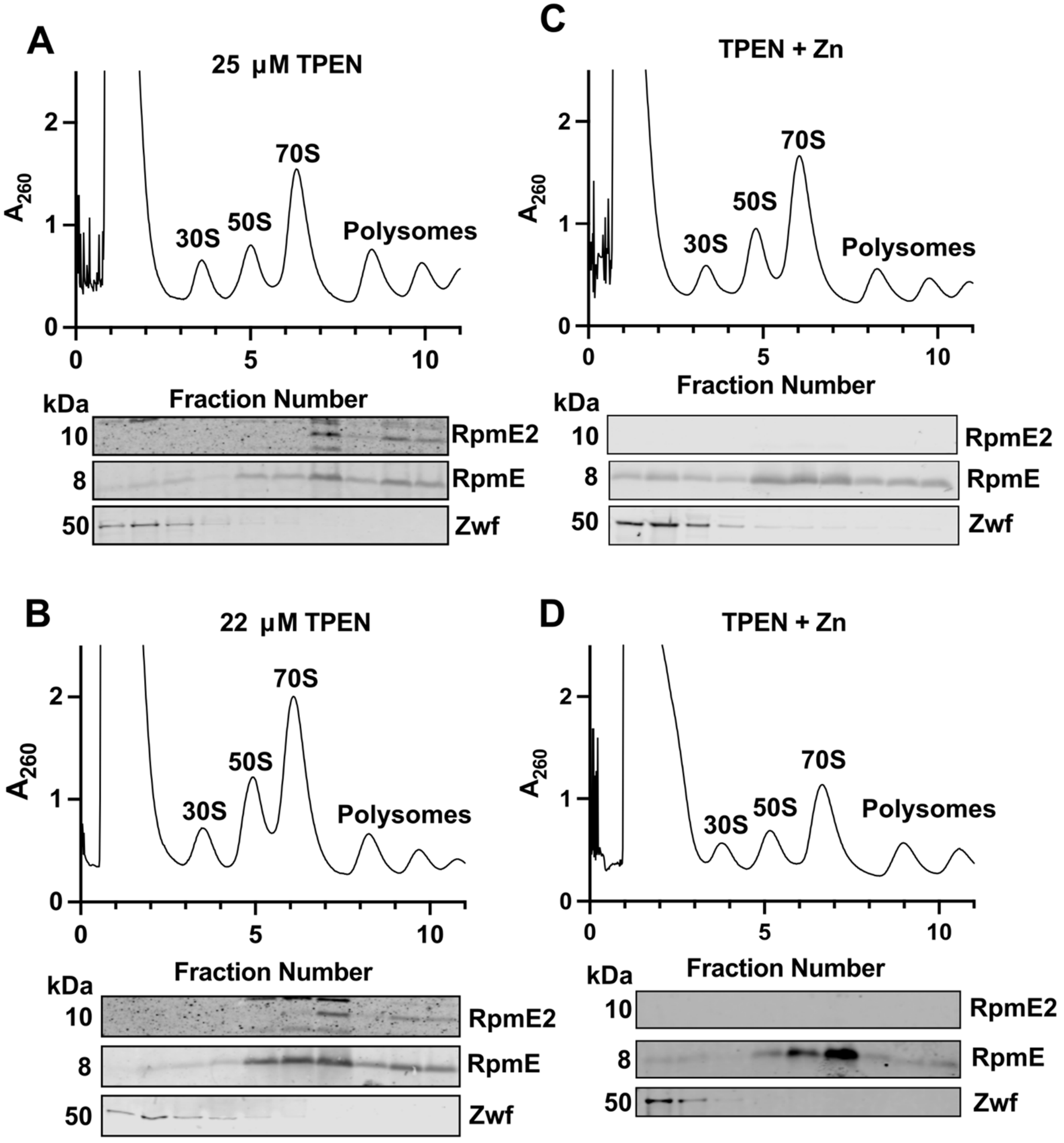

Fig S1. Additional replicates of zinc-limited and zinc-replete polysome profiles, and RpmE2 and RpmE detection in Gc ribosomes. See Fig. 2 for experimental details.

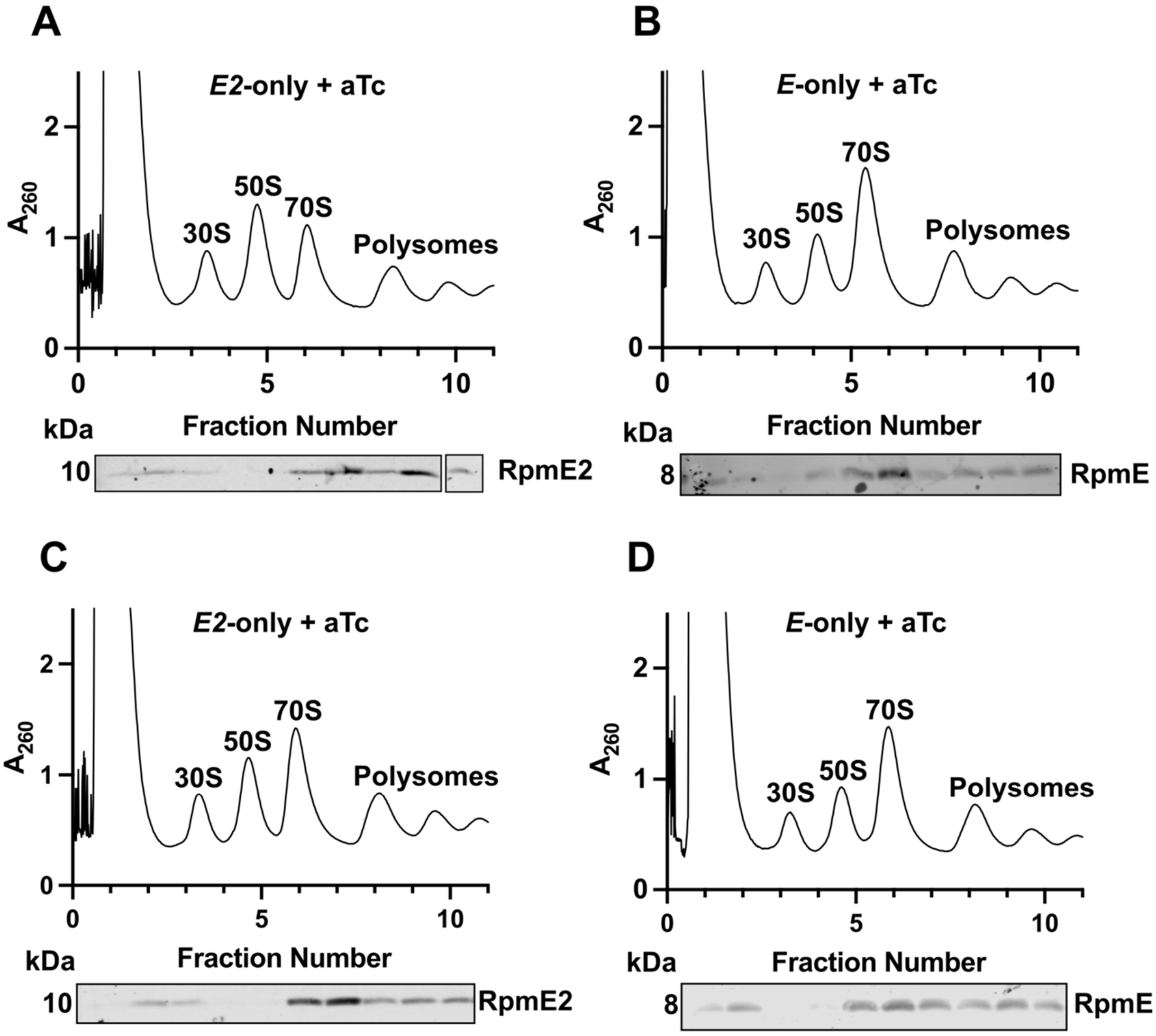

Fig S2. Additional replicates of polysome profiles and immunoblots of *E*-only and *E2*-only Gc ribosomes. See Fig. 4.

```
                      *  *                            *   *
E.coli RpmE2  MKPNIHPE-YRTVVFHDTSVDEYFKIGSTIKTD--REIELDGVTYPYVTIDVSSKSHPFY
Gc RpmE2      MKPNIHPDNYRTVLFFDSSANEGWLIRSCAGTHGKTMVWTDGKEYLLFSLDTSSSSHPVY

E.coli RpmE2  TGKLRTVASEGNVARFTQRFGRFVSTKKGA-
Gc RpmE2      TGKQRNVNTEGRASKFNQRFQSVMSSFRKDK

                               *  *                    *   *
E.coli RpmE   MKKDIHPKYEEITASCSCGNVMKIRSTV-GHDLNLDVCSKCHPFFTGKQRDVATGGRVDR
Gc RpmE       MKQGIHPNYREVNVTCSCGNKFVTKSAMEKENFNIEVCSLCHPFYTGTQKIVDTTGRVDK

E.coli RpmE   FNKRFNIPGSK
Gc RpmE       FNNKFGNLFKR
```

Fig S3. Alignment of Gc RpmE and RpmE2 with their *E. coli* homologs. Asterisks indicate cysteine residues implicated in zinc coordination.

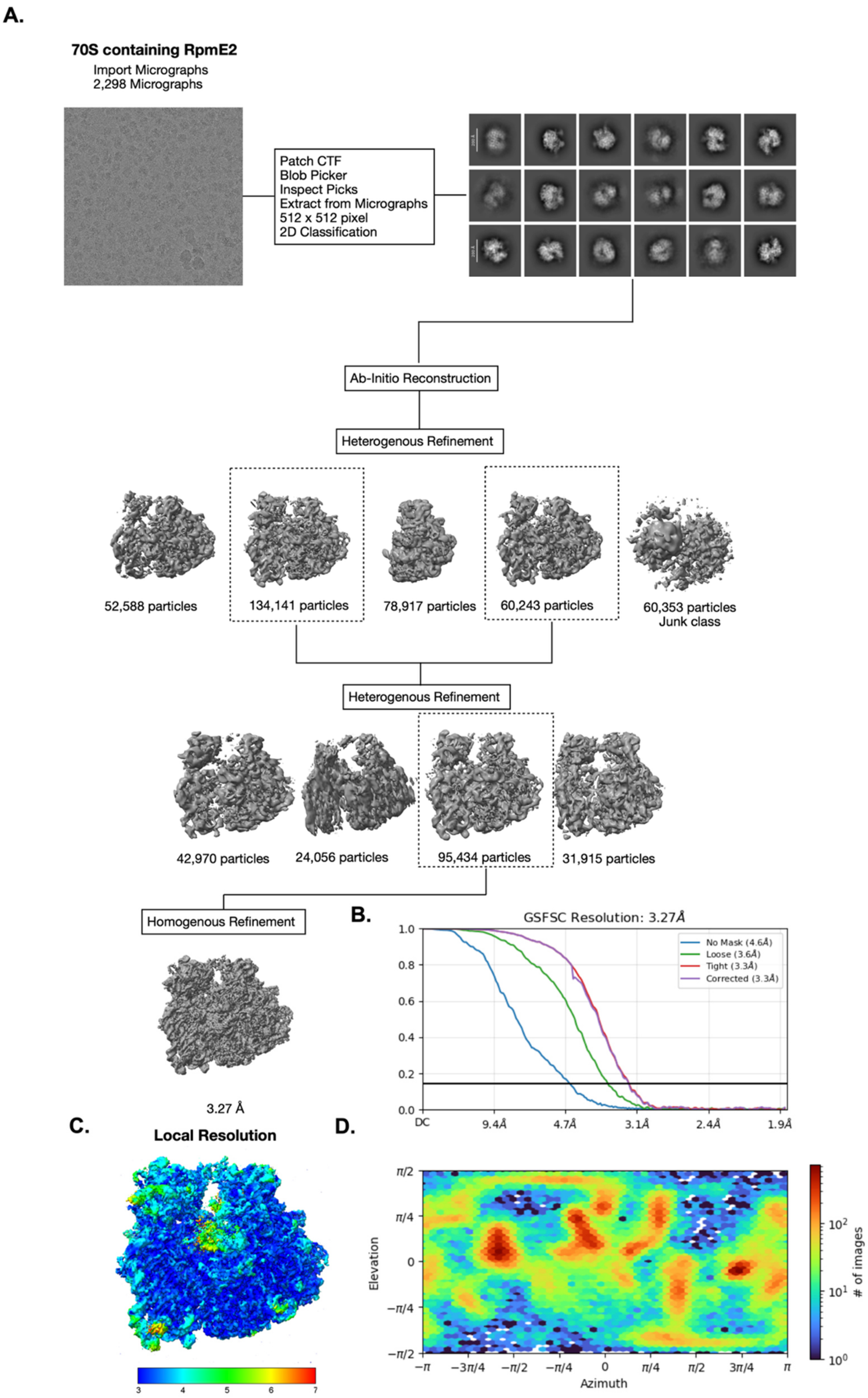
A.
70S containing RpmE2
Import Micrographs
2,298 Micrographs
Patch CTF
Blob Picker
Inspect Picks
Extract from Micrographs
512 x 512 pixel
2D Classification
Ab-Initio Reconstruction
Heterogenous Refinement
52,588 particles
134,141 particles
78,917 particles
60,243 particles
60,353 particles
Junk class
Heterogenous Refinement
42,970 particles
24,056 particles
95,434 particles
31,915 particles
Homogenous Refinement
3.27 Å
B.
GSFSC Resolution: 3.27Å
No Mask (4.6Å)
Loose (3.6Å)
Tight (3.3Å)
Corrected (3.3Å)
1.0
0.8
0.6
0.4
0.2
0.0
DC
9.4Å
4.7Å
3.1Å
2.4Å
1.9Å
C.
Local Resolution
3
4
5
6
7
D.
π/2
π/4
0
−π/4
−π/2
Elevation
−π
−3π/4
−π/2
−π/4
0
π/4
π/2
3π/4
π
Azimuth
10^2
10^1
10^0
# of images

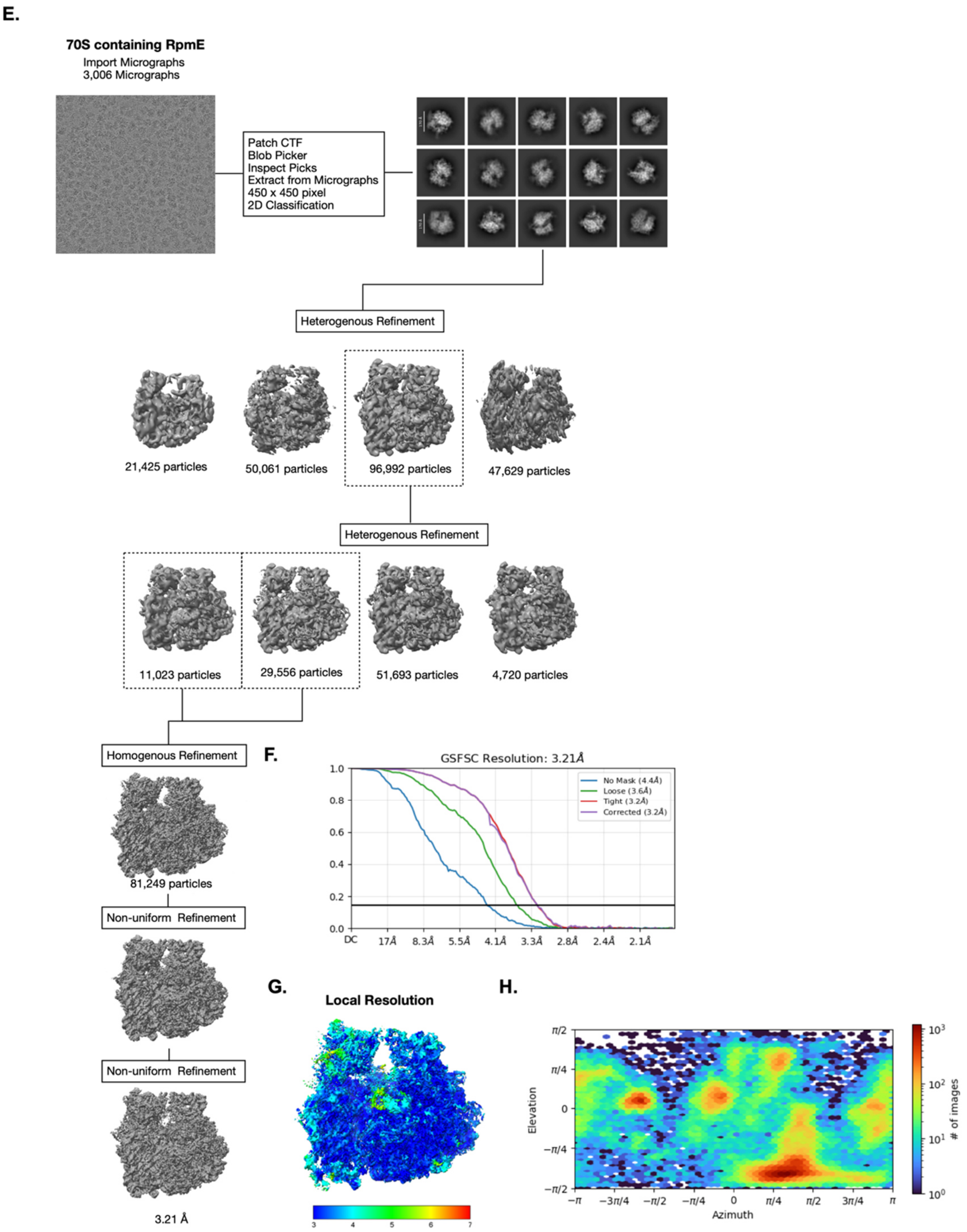
E.
70S containing RpmE
Import Micrographs
3,006 Micrographs
Patch CTF
Blob Picker
Inspect Picks
Extract from Micrographs
450 x 450 pixel
2D Classification
Heterogenous Refinement
21,425 particles
50,061 particles
96,992 particles
47,629 particles
Heterogenous Refinement
11,023 particles
29,556 particles
51,693 particles
4,720 particles
Homogenous Refinement
81,249 particles
Non-uniform Refinement
Non-uniform Refinement
3.21 Å
F.
GSFSC Resolution: 3.21Å
No Mask (4.4Å)
Loose (3.6Å)
Tight (3.2Å)
Corrected (3.2Å)
DC
17Å
8.3Å
5.5Å
4.1Å
3.3Å
2.8Å
2.4Å
2.1Å
G.
Local Resolution
H.
Elevation
Azimuth
# of images

Fig. S4. Processing strategy for 70S ribosomes containing RpmE2 and RpmE. (A, E) Single-particle cryo-EM data processing workflow. Movies were motion-corrected and CTF-estimated prior to particle picking and 2D classification. Particles selected after 2D classification were used to generate an initial consensus reconstruction of the 70S ribosome, followed by homogeneous refinement. (B, F) Global map resolution was estimated by gold-standard Fourier shell correlation (GSFSC) between independently refined half-maps, using the FSC = 0.143 criterion. (C, G) Local resolution distributions calculated in CryoSPARC. (D, H) Angular distribution of particles contributing to the final reconstructions.

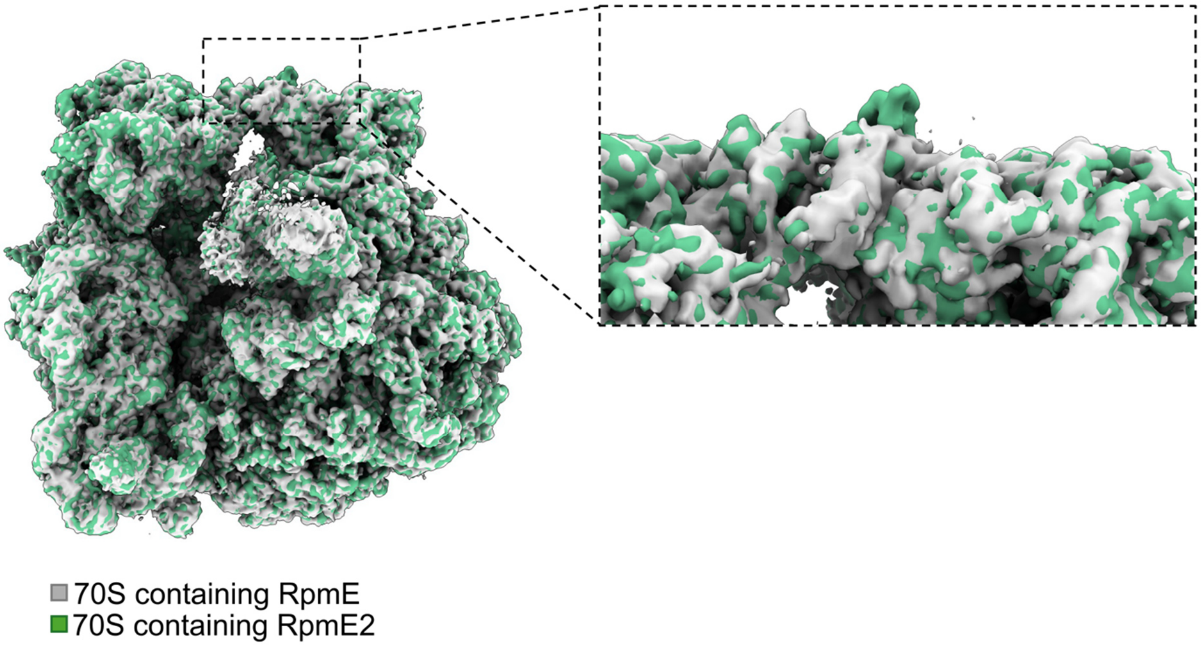

Fig S5. Overlay of density maps from RpmE-only and RpmE2-only ribosomes.

Shown is one orientation of the overlay of the electron density maps from Cryo-EM of RpmE-only (gray) and RpmE2-only (green) Gc 70S ribosomes. The area in the dotted box is shown in greater detail on the right.

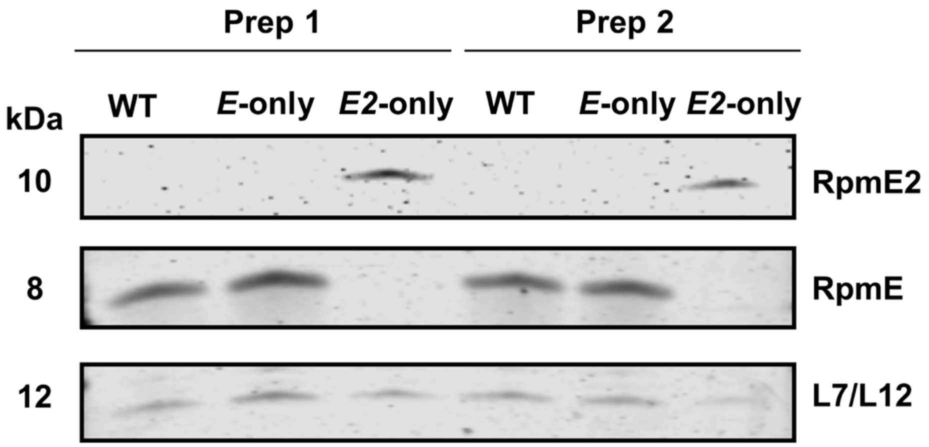

Fig S6. RpmE2 and RpmE are present in ribosomes purified for *in vitro* translation reactions. Sucrose cushion-purified ribosomes from WT, *E*-only, and *E2*-only Gc grown to mid-log phase in modified GCBL + 20 ng/mL aTc were diluted to 17 nM. Protein production was analyzed by 16.5% Tris-Tricine SDS-PAGE, followed by immunoblot for RpmE2, RpmE, and L7/L12 ribosomal protein (loading control).

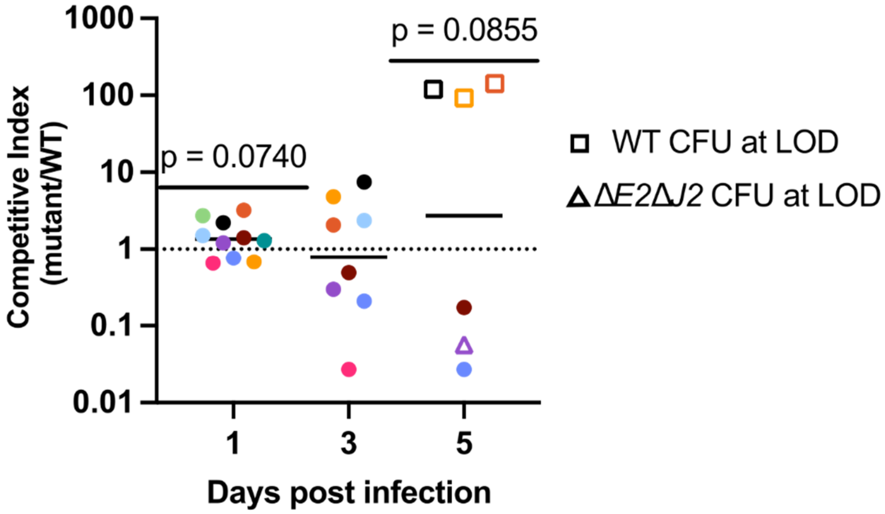

Fig S7. Gc lacking RpmE2 and RpmJ2 exhibit similar fitness to WT *in vivo*. 4-6 week old BALB/c mice were inoculated vaginally with 10^6 CFU of a 1:1 mix of WT and Δ*E2*Δ*J2* Gc. At days 1, 3, and 5 post-infection mice were swabbed vaginally and swabs were sub-cultured on GCB to enumerate CFU. The competitive index (CI) of Δ*E2*Δ*J2* Gc versus WT Gc was calculated by normalizing the ratio of mutant to WT Gc in the output CFU to the ratio of mutant to WT Gc in the input CFU. Each symbol/color represents the CI of 1 mouse and lines represent the geometric mean. The dashed line represents a CI of 1. Open squares represent mice with 0 WT CFU recovered; open triangles represent mice with 0 Δ*E2*Δ*J2* CFU recovered. The limit-of-detection (LOD, 50) was used for mice in which CFU below the LOD for a strain were recovered. Statistics represent the results of a one sample t test relative to the theoretical mean of 1.

Movie S1. Rotated overlay of density maps from RpmE-only and RpmE2-only ribosomes. The electron density maps from Cryo-EM of RpmE-only (gray) and RpmE2-only (green) Gc 70S ribosomes were overlaid and assembled into a movie using ChimeraX (12).

References

1. Stohl EA, Criss AK, Seifert HS. 2005. The transcriptome response of Neisseria gonorrhoeae to hydrogen peroxide reveals genes with previously uncharacterized roles in oxidative damage protection. Mol Microbiol 58:520-32.
2. Anderson JE, Sparling PF, Cornelissen CN. 1994. Gonococcal transferrin-binding protein 2 facilitates but is not essential for transferrin utilization. J Bacteriol 176:3162-70.
3. Ramsey ME, Hackett KT, Kotha C, Dillard JP. 2012. New complementation constructs for inducible and constitutive gene expression in Neisseria gonorrhoeae and Neisseria meningitidis. Appl Environ Microbiol 78:3068-78.
4. Wierzbicki IH, Zielke RA, Korotkov KV, Sikora AE. 2017. Functional and structural studies on the Neisseria gonorrhoeae GmhA, the first enzyme in the glycero-manno-heptose biosynthesis pathways, demonstrate a critical role in lipooligosaccharide synthesis and gonococcal viability. Microbiologyopen 6.
5. Leong V, Kent M, Jomaa A, Ortega J. 2013. Escherichia coli rimM and yjeQ null strains accumulate immature 30S subunits of similar structure and protein complement. RNA 19:789-802.
6. Jomaa A, Stewart G, Martin-Benito J, Zielke R, Campbell TL, Maddock JR, Brown ED, Ortega J. 2011. Understanding ribosome assembly: the structure of in vivo assembled immature 30S subunits revealed by cryo-electron microscopy. RNA 17:697-709.
7. Feaga HA, Kopylov M, Kim JK, Jovanovic M, Dworkin J. 2020. Ribosome Dimerization Protects the Small Subunit. J Bacteriol 202.

8. Jeong H, Barbe V, Lee CH, Vallenet D, Yu DS, Choi SH, Couloux A, Lee SW, Yoon SH, Cattolico L, Hur CG, Park HS, Segurens B, Kim SC, Oh TK, Lenski RE, Studier FW, Daegelen P, Kim JF. 2009. Genome sequences of Escherichia coli B strains REL606 and BL21(DE3). J Mol Biol 394:644-52.

9. UniProt C. 2025. UniProt: the Universal Protein Knowledgebase in 2025. Nucleic Acids Res 53:D609-D617.

10. Raterman EL, Jerse AE. 2019. Female Mouse Model of Neisseria gonorrhoeae Infection. Methods Mol Biol 1997:413-429.

11. Punjani A, Rubinstein JL, Fleet DJ, Brubaker MA. 2017. cryoSPARC: algorithms for rapid unsupervised cryo-EM structure determination. Nature Methods 14:290-296.

12. Pettersen EF, Goddard TD, Huang CC, Meng EC, Couch GS, Croll TI, Morris JH, Ferrin TE. 2021. UCSF ChimeraX: Structure visualization for researchers, educators, and developers. Protein Sci 30:70-82.
